# Comparative pharmacotherapeutic efficacy and dose-related safety profiles of intrathecal baclofen: a systematic review and meta-analysis in neurological spasticity

**DOI:** 10.3389/fphar.2026.1866389

**Published:** 2026-08-24

**Authors:** Haoyuan Chen, Honghui Lei, Yang Yu, Caizhi Ma, Fangyong Wang

**Affiliations:** 1 Department of Spine Surgery, Beijing Bo’ai Hospital, China Rehabilitation Research Center, Beijing, China; 2 School of Rehabilitation, Capital Medical University, Beijing, China; 3 School of Biological Science and Medical Engineering, Beihang University, Beijing, China; 4 University of Health and Rehabilitation Sciences, Qingdao, Shandong, China; 5 Rehabilitation Medicine Center, The Second Affiliated Hospital and Yuying Children’s Hospital, Wenzhou Medical University, Wenzhou, China

**Keywords:** intrathecal baclofen, meta-analysis, safety evaluation, spasm frequency, spasticity

## Abstract

**Background:**

Intrathecal baclofen (ITB) acts as a selective GABA_B_ receptor agonist to modulate spinal inhibitory pathways. While effective for severe spasticity, the pharmacotherapeutic efficacy and safety profile across distinct neurological etiologies—cerebral palsy (CP), spinal cord injury (SCI), and brain injury (BI)—remain poorly characterized from a comparative pharmacological perspective.

**Methods and findings:**

We systematically searched five global databases (up to August 2025) for trials evaluating ITB therapy. Random-effects models were employed to synthesize efficacy data, focusing on dose-response relationships and clinical outcomes. 15 studies (6 RCTs, 9 observational) were analyzed. Our results demonstrate that ITB significantly reduced Ashworth Scale scores across all subgroups, but with marked etiology-specific variations in effective dosage requirements. Specifically, SCI patients exhibited the most significant reduction in spasm frequency. Safety pharmacology analysis revealed that pharmacological adverse events (AEs), such as somnolence and respiratory depression, were dose-dependent and more prevalent in BI populations. In contrast, hardware-related complications (e.g., catheter malfunction) were idiosyncratic but clinically severe.

**Conclusion:**

ITB therapy provides robust anti-spasticity effects mediated through its targeted action on spinal inhibitory circuits. However, the heterogeneity in dosage efficacy suggests etiology-driven differences in GABA_B_ receptor sensitivity or drug distribution. These findings underscore the necessity for precision titration strategies based on patient-specific pathophysiology and highlight the importance of differentiating between pharmacodynamic side effects and mechanical complications to optimize the therapeutic index of ITB.

**Systematic Review Registration:**

https://www.crd.york.ac.uk/PROSPERO/view/CRD420251112388, identifier CRD420251112388.

## 1 Introduction

Spasticity is a common clinical manifestation of upper motor neuron lesions (Lance, 1980; Thibaut et al., 2013) and is frequently observed in conditions such as cerebral palsy (CP) (Sadowska et al., 2020), spinal cord injury (SCI) (Holtz et al., 2017), and brain injury (BI) (Martens et al., 2017). It is characterized by a velocity-dependent increase in tonic stretch reflexes and exaggerated tendon jerks (Lance, 1980), resulting from hyperexcitability of the stretch reflex arc and disinhibition of spinal reflex pathways (Lance, 1980; Trompetto et al., 2014).

In CP, spasticity is commonly attributed to perinatal or early postnatal injury affecting the corticospinal tracts and cortical motor areas (Sadowska et al., 2020; Vitrikas et al., 2020). In SCI, loss of descending inhibitory control from the brainstem and cortex results in segmental hyperexcitability below the lesion level and is often accompanied by maladaptive spinal plasticity (Trompetto et al., 2014; Dietz and Sinkjaer, 2007). In BI, disruption of supraspinal motor pathways and impaired inhibitory control also contribute to the development of spasticity (Thibaut et al., 2013; Urban et al., 2010). Together, these pathophysiological changes can markedly impair daily functioning, social participation, and quality of life (Milinis and Young, 2016; Svensson et al., 2014).

Baclofen, a selective γ-aminobutyric acid type B (GABA_B_) receptor agonist, exerts its antispastic effects primarily at the spinal level (Bowery et al., 1980). It inhibits presynaptic Ca^2+^ influx, thereby reducing the release of excitatory neurotransmitters such as glutamate from primary afferent terminals (Bowery, 1989), and also promotes postsynaptic hyperpolarization of α-motor neurons (Davidoff, 1985). Although oral baclofen is commonly used as a first-line pharmacological treatment, its clinical utility is limited by low lipophilicity and poor penetration across the blood-brain barrier (BBB) (Krach, 2001). Higher systemic doses are therefore often required to achieve therapeutic spinal effects (Krach, 2001), increasing the risk of dose-limiting adverse effects such as somnolence, fatigue, and cognitive impairment (Taricco et al., 2000). This narrow therapeutic window may contribute to suboptimal spasticity control and poor long-term adherence (Krach, 2001; Bhimani and Anderson, 2014).

Intrathecal baclofen (ITB) therapy has emerged as an important therapeutic alternative by circumventing the BBB and delivering baclofen directly into the subarachnoid space (Albright, 1996; Coffey et al., 1993). This targeted delivery achieves effective cerebrospinal fluid (CSF) concentrations at less than 1% of the equivalent oral dose, thereby improving the therapeutic index (Krach, 2001; Coffey et al., 2002). At the spinal level, baclofen suppresses excitatory neurotransmitter release from primary afferent fibers and reduces α-motor neuron excitability (Davidoff, 1985), producing potent antispastic effects while minimizing systemic exposure and associated supraspinal side effects (Bowery, 1989). Accordingly, ITB dosing in clinical practice is individualized and titrated according to patient response and tolerability (Koueik et al., 2025; Boster et al., 2016). Despite these advantages, important clinical controversies remain. A marked reduction in spasticity does not always translate into functional improvement, and excessive pharmacodynamic inhibition may compromise postural stability (Maneyapanda et al., 2017). In addition, safety assessments often combine pharmacological adverse events with hardware-related complications, potentially obscuring their distinct clinical implications (Ryan et al., 2024; Koueik et al., 2025).

However, an important knowledge gap remains regarding whether the efficacy and safety profile of ITB differs across etiologies such as CP, SCI, and BI (Boster et al., 2016). Differences in underlying pathophysiology, lesion characteristics, and neural circuit reorganization may contribute to variations in treatment response and adverse event patterns (Trompetto et al., 2014). Therefore, this meta-analysis aimed to quantitatively evaluate the etiology-specific effects of ITB therapy on spasticity, motor function, pharmacological adverse events, and hardware-related complications. These findings may help inform individualized treatment strategies and improve the balance between therapeutic benefit and treatment-related risk.

## 2 Materials and methods

This study was registered with International Prospective Register of Systematic Reviews (PROSPERO) on 25 July 2025. A secondary expanded literature search was conducted on 4 September 2025, followed by a major revision of the registration details on 9 February 2026, which primarily involved adjustments to the Participants, Interventions, Comparisons, Outcomes, and Study design (PICOS) criteria, as well as the primary objectives and outcomes of this systematic review (CRD420251112388). The methodology was rigorously conducted in accordance with the Cochrane Handbook to ensure methodological robustness, and the reporting adhered to the Preferred Reporting Items for Systematic Reviews and Meta-Analyses (PRISMA) guidelines to guarantee comprehensiveness and transparency. The full study protocol is available at https://www.crd.york.ac.uk/PROSPERO/view/CRD420251112388.

### 2.1 Literature search

Adhering to PRISMA guidelines, two researchers independently searched the PubMed, Web of Science, Cochrane Library, Scopus, and China National Knowledge Infrastructure databases. The initial search (up to August 2025) focused on randomized controlled trials (RCTs). Given the limited volume of relevant literature and data, the scope was subsequently expanded to include single-arm trials (SATs), with the search updated through September 2025 (Supplementary Material: Search Strategy). To ensure the integrity of data extraction and sufficient statistical power, studies involving stroke and multiple sclerosis were excluded due to missing numerical values and insufficient sample sizes for analysis. The final analysis focused on three cohorts with adequate data: CP, BI, and SCI. The search results were cross-checked by both researchers until full consensus was reached.

Two researchers independently performed preliminary screening and full-text review of the citations using EndNote software based on established criteria (Table 1). The screening results were cross-checked by both researchers; any discrepancies were resolved through consultation with a third researcher. In this study, full consensus was reached between the two researchers, and no arbitration procedure was required.

**TABLE 1:** Inclusion and exclusion criteria for the systematic review: Based on the PICOS principle.

Criteria (PICOS)	Description
Population (P)	Patients diagnosed with neurological disorders, including spinal cord injury, brain injury (e.g., traumatic brain injury, cerebrovascular disease), and cerebral palsy. Baseline spasticity must meet the criterion of a modified ashworth scale or ashworth scale score ≥1
Intervention (I)	Inclusion: Intrathecal baclofen therapy, administered via continuous infusion or intermittent bolus Exclusion: Non-intrathecal administration routes, acute spasticity states, animal studies, and case reports
Comparison (C)	Conventional therapy, pharmacological treatment, physical therapy, sham stimulation, or other non-specified interventions
Outcomes (O)	Primary outcomes: For spinal cord injury and brain injury: Modified ashworth scale or ashworth scale scores; spasm frequency scale results For cerebral palsy: Modified ashworth scale or ashworth scale scores; gross motor function measure Assessment timepoints Immediate effect: Time interval between baseline and post-intervention assessment ≤24 h Long-term effect: Time interval between baseline and post-intervention assessment ≥4 weeks
	Secondary outcomes: 1. Activities of daily living assessments, including modified barthel index, etc. (data limited to timepoints ≥4 weeks post-intervention) 2. For cerebral palsy: Functional improvement reflected by nursing difficulty assessments, including ease of care scale 3. All reported adverse eventss during the intrathecal baclofen intervention period to evaluate safety
Study design (S)	Randomized controlled trials and single arm trials Exclusion criteria: Randomized controlled trials with a sample size ≤10; single arm trials with a sample size ≤5; follow-up duration outside the predefined range; studies with incomplete or non-extractable data

### 2.2 Data extraction and outcome assessment

Data were independently extracted by three researchers working in pairs, with any discrepancies resolved through collective discussion or adjudication by a senior researcher. The extracted data included: baseline and clinical characteristics (author, year, study design, sample size, age), pharmacological parameters (mean daily ITB maintenance dosage) and outcome measures: Efficacy indicators (e.g., Ashworth Scale (AS) scores, spasm frequency scale (SFS)) and safety pharmacology profiles. To maximize statistical power despite the limited number of original RCTs, data from ITB intervention groups in RCTs were pooled with SATs for analysis. Safety outcomes were specifically categorized into pharmacological adverse events (AEs) (Pharmacological Adverse Events (PAEs)) and hardware-related complications (Catheter-Related Adverse Events (CRAEs); Pump-Related Adverse Events (PRAEs); Surgery-Related Adverse Events (SRAEs)) to differentiate drug-induced effects from mechanical failures. For missing key data (mean/standard deviation), we either contacted the original authors or utilized WebPlotDigitizer software to extract and supplement data from published figures.

### 2.3 Risk of bias assessment

This study employed different assessment tools based on the specific study designs. For RCTs units, risk of bias was primarily assessed using the Cochrane Risk of Bias tool, version 2.0 (RoB 2.0). For SATs, the Risk Of Bias In Non-randomized Studies of Interventions tool (ROBINS-I) was utilized. These assessments were performed independently by three researchers, with any discrepancies adjudicated by a third researcher. For mixed-design studies, we disaggregated them into independent analytical units. If a study reported data from both an RCT phase and a subsequent SAT phase, the corresponding tools were applied separately for quality assessment and data extraction. If only one phase met the inclusion criteria, the assessment was limited to that specific phase.

### 2.4 Data synthesis and statistical analysis

Meta-analysis was performed using R software (version 4.5.1). Considering the potential heterogeneity in study populations (adults vs. children) and clinical characteristics (e.g., disease etiology, titration protocols). All effect sizes were pooled using a random effects model to ensure the robustness of the results. Modular analyses were conducted by categorizing studies into three subgroups based on etiology: CP, SCI, and BI. For mixed-design studies, we disaggregated them into independent analytical units to account for the heterogeneity of study designs during statistical pooling. Consequently, there is a discrepancy between the number of analytical units included in the meta-analysis (n = 18) and the number of original publications (n = 15).

For dichotomous variables (AEs), risk ratios with 95% confidence interval (CI) were primarily employed for analysis. For continuous variables, mean difference (MD) was used if the measurement tools were identical; otherwise, standardized mean difference (SMD) was applied. Heterogeneity was assessed using the I^2^ statistic, where I^2^ ≤ 40 indicated low, 40% < I^2^ ≤ 60% moderate, and 60% < I^2^ ≤ 90% high heterogeneity. To explore the sources of potential heterogeneity within each condition, subgroup analyses will be pre-specified based on the following criteria: duration of intervention, anatomical site of intervention, dosage titration protocols, and etiological subtypes.

To ensure the robustness of the primary findings, sensitivity analyses were performed using the “leave-one-out” method. For multiple publications originating from the same study population, only the single most comprehensive study was extracted to avoid sample duplication. Given that each subgroup included fewer than 10 studies, funnel plots and Egger’s tests were not performed to assess publication bias. Statistical significance was defined as P < 0.05 for all analyses in this study.

### 2.5 Data availability

As this is a systematic review, all data supporting the findings of this study are available within the published articles cited in the reference list. No new primary data were generated for this study.

## 3 Results

**FIGURE 1:**
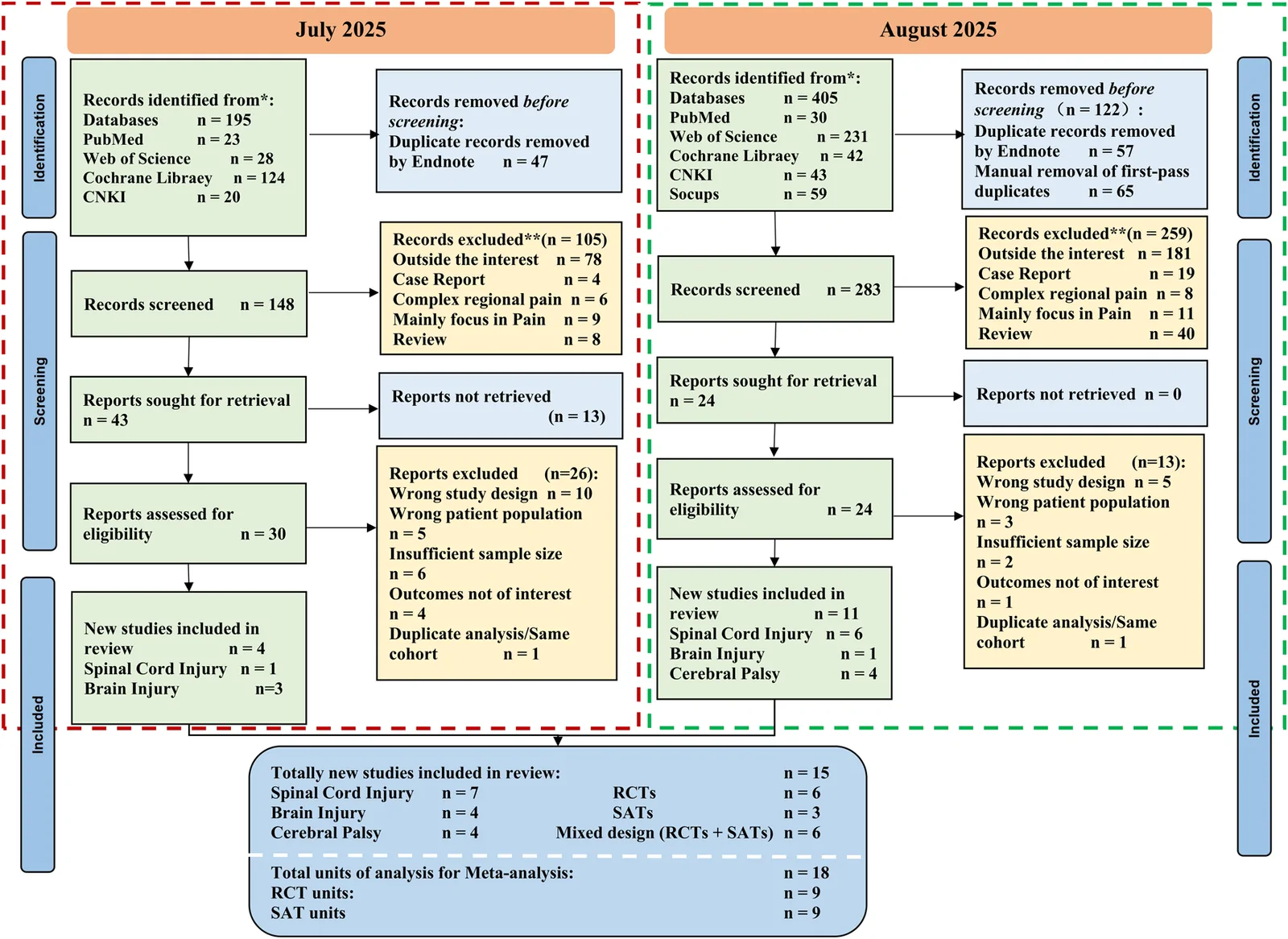
Literature Screening Flowchart. Legend: PRISMA, Preferred Reporting Items for Systematic Reviews and Meta-Analyses (Based on the PRISMA Statement). CNKI: China National Knowledge Infrastructure; RCTs: Randomized controlled trails; SATs: Single-arm trails.

The literature search and selection process is illustrated in Figure 1 The initial searches conducted in July and August 2025 yielded a total of 600 records. After removing duplicates and studies that did not meet the eligibility criteria, 15 independent original publications were ultimately included. Given that 6 studies utilized a mixed-methods design, they were disaggregated into independent analytical units to ensure statistical rigor. This resulted in 18 analytical units (comprising 9 RCT units and 9 SAT units) for inclusion in the meta-analysis. Detailed information regarding the included studies is presented in Table 2.

**TABLE 2:** Characteristics of included studies.

Study ID	Published year	Disease	Design & unit	Simple	Intervention and control		Age (group)	Outcome measures
Subgroup I: Spinal cord injury								
Middel B, et al.	1997	Spinal cord injury	Randomized controlled trials Randomized controlled trial unit	13	Intrathecal baclofen bolus (n = 8)	Placebo (n = 5)	48.5 (adults)	Ashworth scale Spasm frequency score Self report scale
Kumru H, et al.	2018	Spinal cord injury	Single arm trials Single-arm trial unit	18	Continuous intrathecal baclofen		38.5 (adults)	Modified ashworth scale Numerical rating scale
Ordia JI, et al.	2002	Spinal cord injury	Single arm trials Single-arm trial unit	133	Continuous intrathecal baclofen		42 (adults)	Ashwoth scale Spasm frequency score Safety outcomes
Azouvi P, et al.	1996	Spinal cord injury	Randomized controlled trials Randomized controlled trial unit	22	Intrathecal baclofen bolus (n = 12)	Placebo (n = 10)	Intrathecal baclofen: 48.5 Placebo: 46.3 (adults)	Ashworth scale Spasm frequency score Activities of daily living Baclofen dosage Safety outcomes
Meythaler, J. M., et al.	1992	Spinal cord injury	Single arm trials Single-arm trial unit	10	Continuous intrathecal baclofen		24∼62 (adults)	Ashworth scale Spasm frequency score Baclofen dosage Safety outcomes
Penn, et al.^a^	1989	Spinal cord injury	Mixed study Single-arm trial unit	20	Continuous intrathecal baclofen		None	Ashworth scale Spasm frequency score Baclofen dosage Safety outcomes
Coffey JR, et al.	1993	Spinal cord injury	Mixed study Randomized controlled trial unit	186	Intrathecal baclofen bolus (n = 93)	Placebo (n = 93)	40.2 (adults) 42.1 (adults)	Ashworth scale Spasm frequency score Baclofen dosage Safety outcomes
			Mixed study Single-arm trial unit	75	Continuous intrathecal baclofen			
Subgroup II: Brain injury								
Meythaler JM, et al.	1996	Brain injury	Randomized controlled trials Randomized controlled trial unit	22	Intrathecal baclofen bolus (n = 11)	Placebo (n = 11)	25 (adults)	Ashworth scale Spasm frequency score
Meythaler JM, et al.	2001	Cerebrovascular accident	Mixes study Randomized controlled trial unit	42	Intrathecal baclofen bolus (n = 21)	Placebo (n = 21)	50 (adults) 50 (adults)	Ashworth scale Spasm frequency scale Safety outcomes
			Mixed study Single-arm trial unit	17	Continuous intrathecal baclofen			
Creamer M, et al.	2018	Cerebrovascular accident	Randomized controlled trials Randomized controlled trial unit	48	Intrathecal baclofen bolus (n = 24)	Conventional medical treatment (n = 24)	Intrathecal baclofen: 56.1 Placebo: 55.7 (adults)	Ashworth scale Numerical rating scale
Meythaler JM, et al.^a^	1999	Brain injury	Mixed study Single-arm trial unit	17	Continuous intrathecal baclofen		29 (adults)	Ashworth scale Spasm frequency scale Safety outcomes
Subgroup III: Cerebral palsy								
Hoving MA, et al.	2007	Cerebral palsy	Randomized controlled trials Randomized controlled trial unit	27	Intrathecal baclofen bolus (n = 14)	Placebo (n = 13)	13Y2M^b^ (children)	Ease of care Safety outcomes
Hoving MA, et al.	2007	Cerebral palsy	Randomized controlled trials Randomized controlled trial unit	12	Intrathecal baclofen bolus (n = 7)	Placebo (n = 5)	13Y7M^b^ (children)	Gross motor function Measure-66 Ease of care
Van schaeybroeck P, et al. ^a^	2000	Cerebral palsy	Mixed study Single-arm trial unit	6	Continuous intrathecal baclofen		15–24 (Adults + Children)	Ashworth scale Safety outcomes
Gilmartin R, et al.	2000	Cerebral palsy	Mixed study Randomized controlled trial unit	44	Intrathecal baclofen bolus (n = 22)	Placebo (n = 22)	10.3 (children)	Ashworth scale Baclofen dosage Safety outcomes
			Mixed study Single-arm trial unit	41	Continuous intrathecal baclofen		None	

^a^ indicates that although the included study was a mixed study, for various reasons (such as inconsistent outcome measures), only data from one phase of the trial (which could be either an Randomized Controlled Trial or an Single-Arm Trial) was included.

^b^ indicates that the mean age of participants in the included study is expressed in the format of ‘X years X month.

### 3.1 Characteristics of included studies

The 15 included original publications were published between 1992 and 2018. Seven studies focused on adult SCI (Middel et al., 1997; Kumru et al., 2018; Ordia et al., 2002; Azouvi et al., 1996; Coffey et al., 1993; Meythaler et al., 1992; Penn Richard et al., 1989), four on adult BI (Creamer et al., 2018; Meythaler et al., 1996; Meythaler et al., 1999; Meythaler et al., 2001), and four on pediatric CP (Gilmartin et al., 2000; Van Schaeybroeck et al., 2000; Hoving et al., 2007; Hoving et al., 2009). A total of 753 subjects were included across the studies. Among these, the RCT units comprised 416 participants (ITB group, n = 212; control group, n = 204), while the SAT units included 337 participants. The detailed demographic characteristics, study designs, and outcome measures of all included studies are summarized in Table 2.

### 3.2 Methodological quality and risk of bias assessment

RoB 2.0 assessments (Supplementary Material: Supplementary Figures S1a,b) identified “some concerns” (77.78%) in most RCT and “high risk” (22.22%) in the remainder. High-risk RCT were concentrated in the SCI subgroup (two-thirds of the group), limiting evidence robustness. All SAT were judged as “high risk” via ROBINS-I (Supplementary Material: Supplementary Figures S1c,d), primarily due to single-arm designs and lack of blinding (Domains 1 and 6). Consequently, ITB efficacy results require cautious interpretation due to the overall high risk of bias.

### 3.3 Spinal cord injury

#### 3.3.1 Pooled analysis of Ashworth Scale scores

##### 3.3.1.1 Overall results and heterogeneity

In the SCI population, ITB therapy significantly reduced AS scores (Supplementary Material: Supplementary Figure S2). However, high heterogeneity was observed.

##### 3.3.1.2 Subgroup analysis results

**FIGURE 2:**
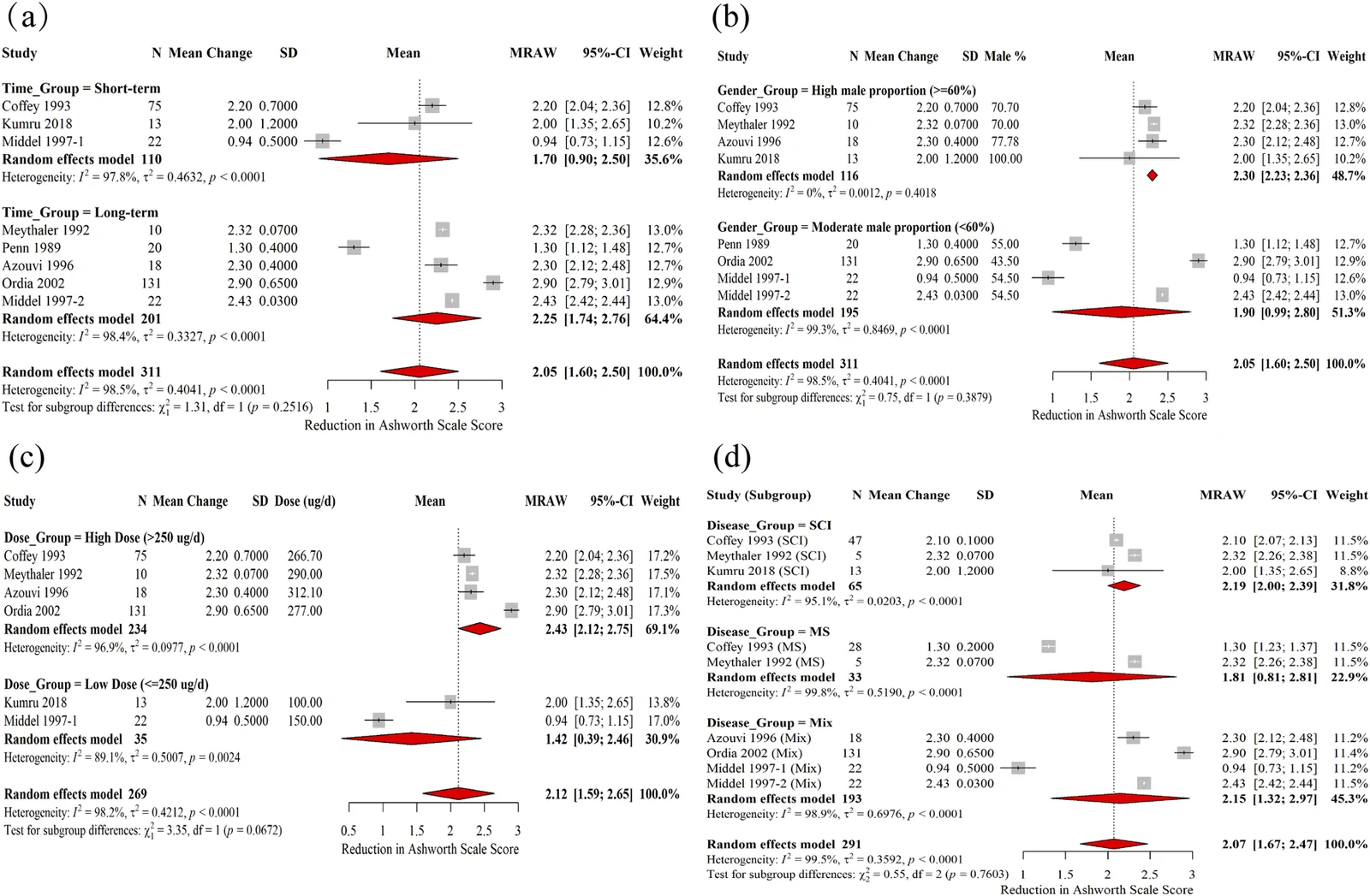
Subgroup analysis of intrathecal baclofen-related efficacy profiles. Legend: **(a)** Follow-up Duration: Long (≥6 months) vs. Shot (<6 months) **(b)** Gender: Comparison of different male proportions **(c)** Dosage: High (>250 μg/day) vs. Low (≤250 μg/day) **(d)** Disease: Comparison between spinal cord injury, Multiple Sclerosis (MS), and Mix (spinal cord injury + MS). SD, Standard difference; MRAW, Mean raw; CI, Confidence interval.

Etiological analysis (Figure 2d) confirmed ITB efficacy across SCI, multiple sclerosis, and mixed populations. While subgroup differences were non-significant, the SCI group demonstrated exceptional homogeneity, indicating high therapeutic predictability.

Regarding duration (Figure 2a), significant improvements were maintained across both short-term and long-term follow-ups, suggesting longitudinal stability of the antispasmodic effect.

Dosage analysis (Figure 2c) revealed consistent therapeutic efficacy across both high- and low-dosage cohorts. The lack of a significant inter-group difference suggests that ITB achieves a robust pharmacodynamic response even at lower dose thresholds, effectively saturating the targeted spinal GABA_B_ receptors.

Furthermore, gender ratio had no significant impact on treatment response (Figure 2b).

##### 3.3.1.3 Sensitivity analysis

Sensitivity analyses (Supplementary Material: Supplementary Figure S3) confirmed the stability of the primary outcomes. Leave-one-out analysis indicated that no individual study disproportionately influenced the pooled results. After excluding potential outliers in high-heterogeneity subgroups (Middel et al., 1997; Ordia et al., 2002), the direction of the therapeutic effect remained unchanged, reinforcing the overall robustness of the findings.

#### 3.3.2 Pooled analysis of spasm frequency scale scores

##### 3.3.2.1 Overall results and heterogeneity

**FIGURE 3:**
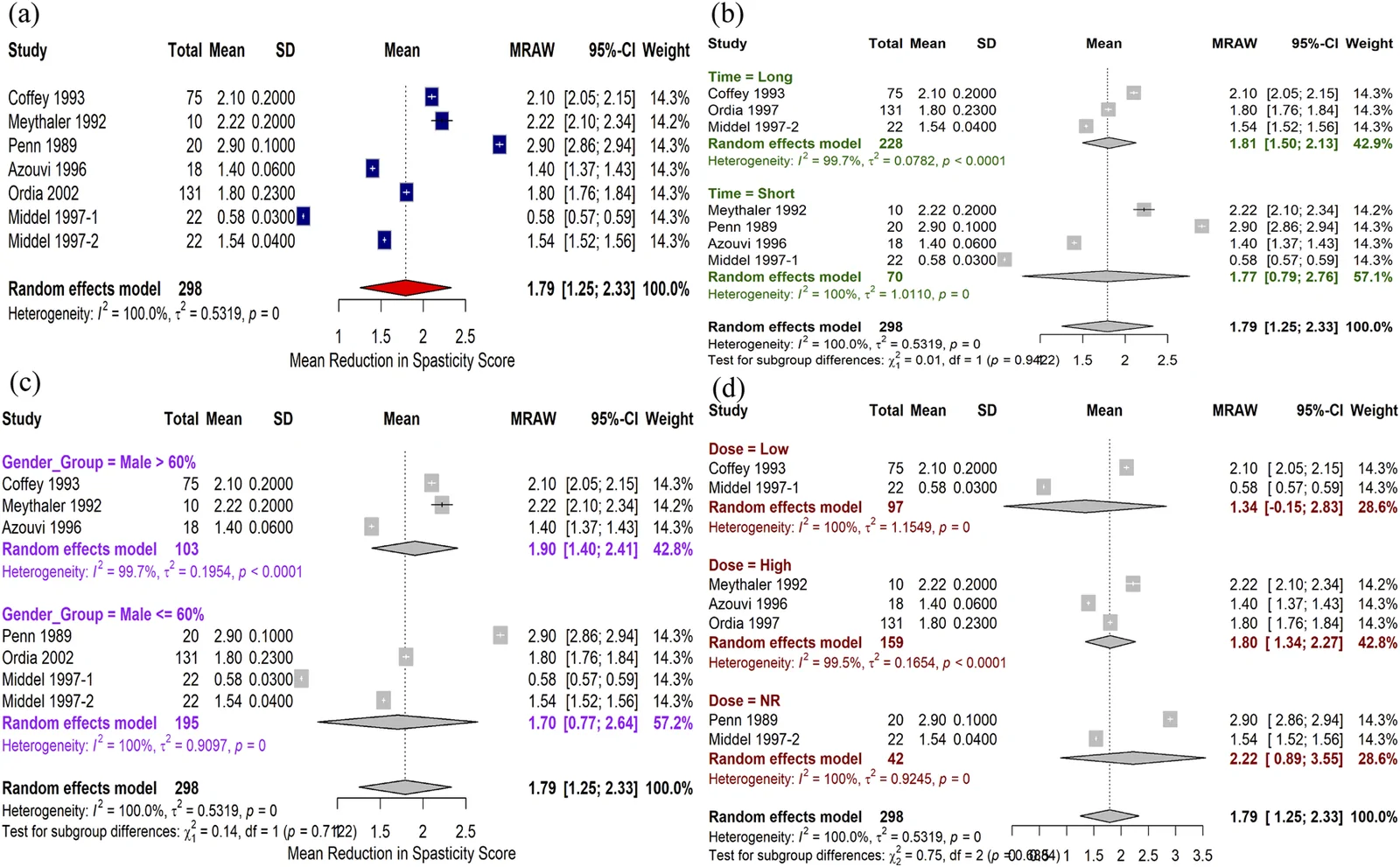
Intrathecal baclofen therapy on spasticity frequency. Legend: **(a)** Meta-analysis of the overall effect of intrathecal baclofen therapy on Spasticity Frequency Score. **(b)** Follow-up Duration: Long (≥6 months) vs. Shot (<6 months). **(c)** Gender: Comparison of different male proportions. **(d)** Daily: High (>250 μg/day) vs. Low (≤250 μg/day). SD, Standard difference; MRAW, Mean raw; CI, Confidence interval.

Analysis of SFS scores (Figure 3a) indicated that ITB therapy significantly reduced spasm frequency in this population, although substantial heterogeneity was observed across the included studies.

##### 3.3.2.2 Subgroup analysis results

Analysis of follow-up duration (Figure 3b) demonstrated significant improvements in SFS scores at both short-term and long-term intervals, with no significant difference between groups, suggesting stable longitudinal efficacy. Similarly, dosage level analysis (Figure 3d) indicated consistent clinical benefits regardless of high or low dosages, with no statistically significant dosage-response relationship. Gender ratio analysis (Figure 3c) further confirmed that therapeutic effects remained consistent across studies, indicating an absence of gender bias in treatment response.

##### 3.3.2.3 Sensitivity analysis

Sensitivity analysis confirmed the stability and robustness of the pooled effect sizes. No individual study exerted a disproportionate influence on the overall results, reinforcing the clinical conclusions.

#### 3.3.3 Multiple comparisons of adverse events

##### 3.3.3.1 Pharmacological adverse events

PAEs were predominantly characterized by gastrointestinal and central nervous system (CNS) effects. Constipation was the most prevalent PAEs, followed by headache, somnolence, and hypotension (Table 3). Somnolence and respiratory depression were observed as CNS–related adverse events and may be associated with higher dose exposure. While most PAEs were transient and manageable via pharmacological titration, severe manifestations such as seizures necessitated immediate cessation of therapy.

**TABLE 3:** The analysis of pharmacological adverse events and associated mechanisms in spinal cord injur**y**.

Type of adverse event	No. of studies	Total patients	Pooled incidence (%)	95% CI	I^2^	Proposed pharmacological mechanism
Constipation	2	141	9.93	5.97; 16.70	13.1%	Inhibition of excitatory neurotransmitter release in the enteric nervous system via GABA_B_ receptors
Urinary retention	2	141	3.55	1.48; 8.24	13.9%	Suppression of the parasympathetic outflow and detrusor muscle contraction reflex at the spinal level
Headache	2	141	4.96	2.39; 10.05	0.0%	Compensatory cerebrovascular autoregulation or transient fluctuations in CSF pressure
Drowsiness	3	151	3.97	1.80; 8.56	82.6%	Non-selective activation of supraspinal GABA_B_ receptors in the brainstem and cortex
Hypotension	3	151	3.97	1.80; 8.56	82.2%	Inhibition of sympathetic preganglionic neurons in the spinal cord, leading to reduced peripheral vascular resistance

Legend: Mechanisms are proposed based on the known pharmacodynamic profile of baclofen as a selective GABA_B_, receptor agonist. CSF: cerebrospinal fluid; GABA_B_: γ-aminobutyric acid type B.

##### 3.3.3.2 Device-related adverse events

**FIGURE 4:**
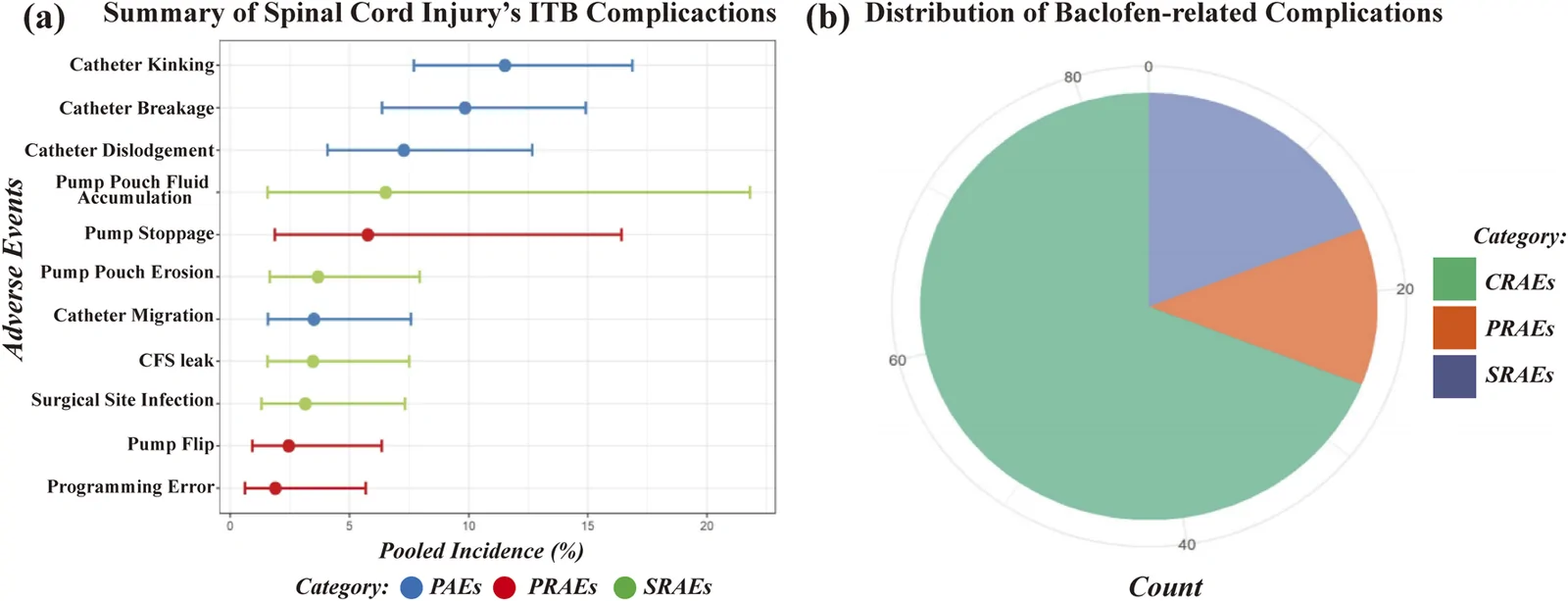
**(a)** Pooled incidence (%) of individual adverse events, grouped into PAEs, PRAEs, and SRAEs. Points indicate the pooled incidence estimates, and horizontal error bars represent the corresponding confidence intervals. **(b)** Distribution of the total number of reported baclofen-related complications according to complication category.

Non-Pharmacological AEs were categorized into catheter-related, pump-related, and surgery-related events (Figure 4; Supplementary Material: Supplementary Table SI). Among CRAEs, catheter kinking was the most frequent, followed by breakage and dislodgement. Pump stoppage was the primary PRAEs, while pump flip and programming errors were rare. For SRAEs, pump pouch fluid accumulation and erosion were the most common manifestations. SRAEs, including implantation site pain (Penn Richard et al., 1989), mortality, and bacterial meningitis (Ordia et al., 2002), were also documented in individual studies.

### 3.4 Brain injury

#### 3.4.1 Pooled analysis of Ashworth scale scores

##### 3.4.1.1 Overall results and heterogeneity

In the RCT units (Supplementary Material: Supplementary Figure S4a), ITB treatment significantly reduced AS scores compared to baseline. Despite moderate heterogeneity, the direction of effect across all trials remained consistent, supporting the clinical superiority of ITB. In the SAT units (Supplementary Material: Supplementary Figure S4b), pooled post-treatment scores exhibited high homogeneity, further confirming the therapeutic efficacy and reproducibility of ITB in maintaining lower muscle spasticity levels within clinical practice.

##### 3.4.1.2 Subgroup analysis results

**FIGURE 5:**
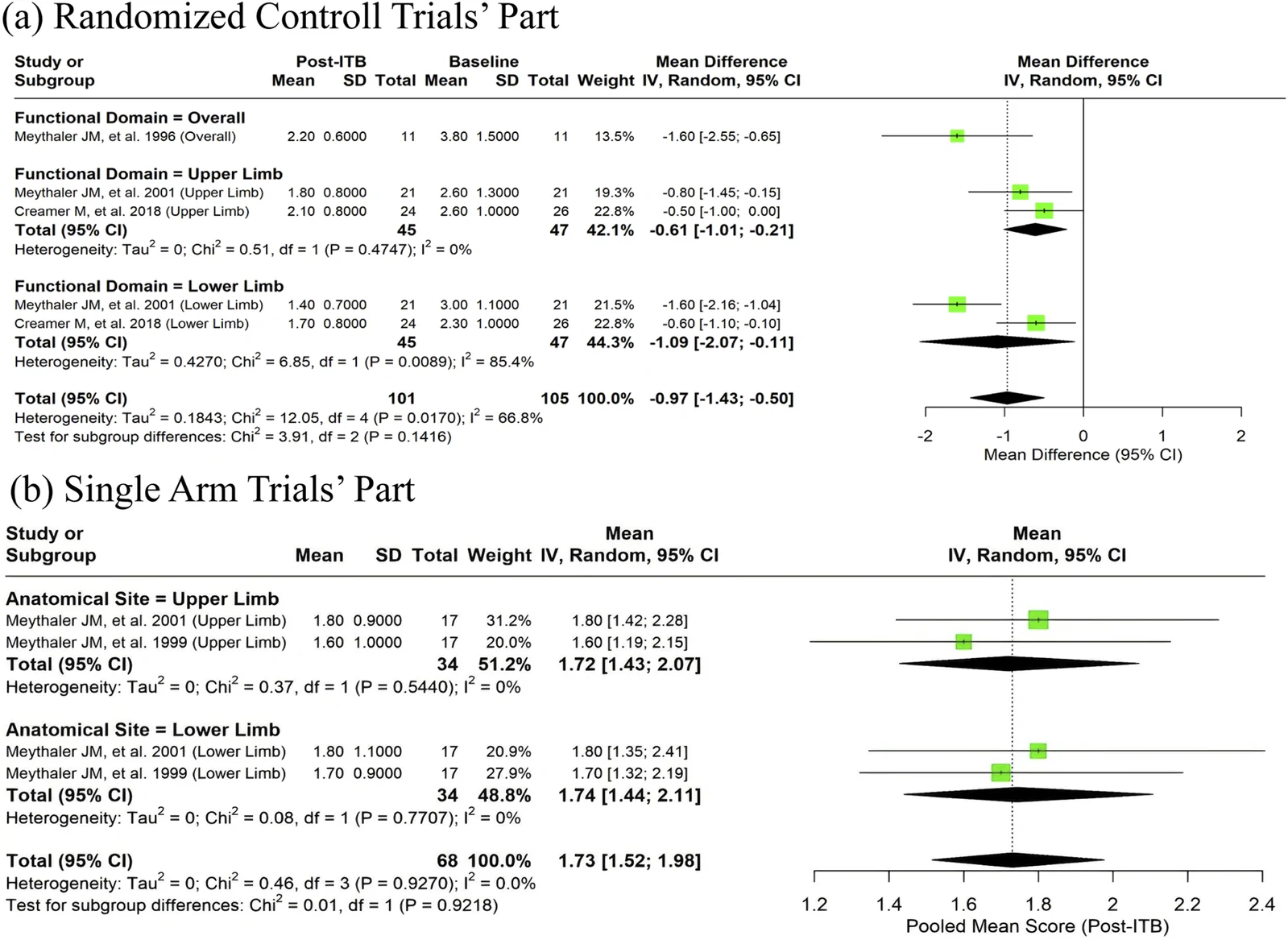
Subgroup analysis of intrathecal baclofen therapy on Ashworth Scale in Brain Injury. Legend: **(a)** Meta-analysis of the Functional Domain subgroup of intrathecal baclofen therapy of randomized controlled trials. **(b)** Meta-analysis of the Functional Domain subgroup of intrathecal baclofen therapy of single arm trials. SD, Standard difference; IV, inverse variance.

In the RCT units (Figure 5a), ITB treatment demonstrated significant reductions in AS scores for both upper and lower limbs. While no statistical difference was observed between these groups, upper limb studies exhibited higher homogeneity. Similarly, in the SAT units (Figure 5b), scores for upper and lower limbs were comparable, with low heterogeneity observed across groups. However, due to the limited number of studies in these subgroups, these findings require cautious interpretation and further validation through large-sample research.

##### 3.4.1.3 Sensitivity analysis

Leave-one-out sensitivity analysis (Supplementary Material: Supplementary Figure S5) confirmed the robustness of the pooled effect size. The exclusion of a single outlying study substantially reduced heterogeneity, identifying it as the primary source of variance. Nevertheless, the effect size remained consistent across analyses, supporting the reliability of ITB in reducing spasticity scores.

#### 3.4.2 Pooled analysis of spasm frequency scale scores

##### 3.4.2.1 Overall results and heterogeneity

In the RCT units (Supplementary Material: Supplementary Figure S6a), ITB treatment significantly reduced SFS scores compared with baseline. Similarly, SAT unit analysis (Supplementary Material: Supplementary Figure S6b) demonstrated that muscle spasticity was maintained at lower levels following treatment. High heterogeneity was observed across both study designs.

##### 3.4.2.2 Subgroup analysis results

**FIGURE 6:**
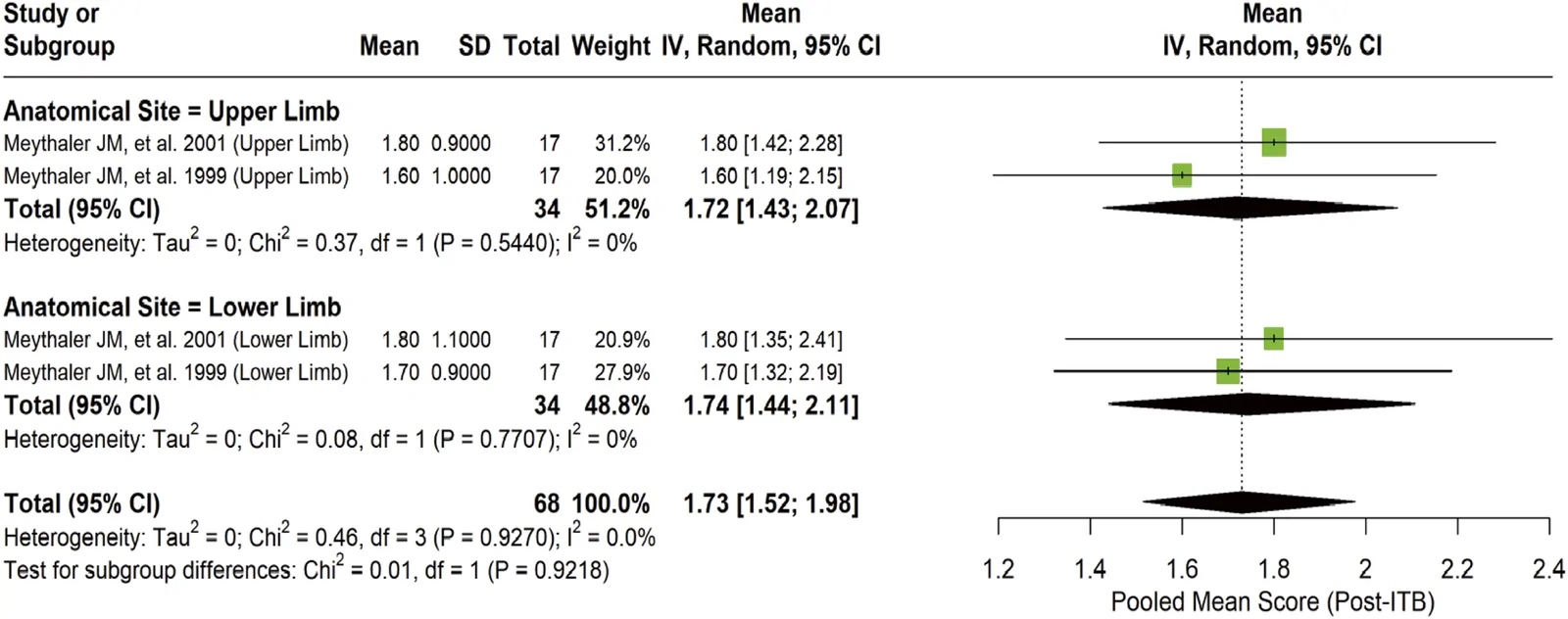
Subgroup analysis of intrathecal baclofen therapy on Spasticity Frequency in Brain Injury SD: standard difference; IV: inverse variance.

Due to the small sample size, subgroup analysis was not performed for RCT units. In the SAT units (Figure 6), ITB treatment showed consistent efficacy for upper limb spasticity with high homogeneity. In contrast, while beneficial, the efficacy for lower limb spasticity demonstrated significant heterogeneity across studies. No statistically significant difference was observed between the upper and lower limb subgroups.

##### 3.4.2.3 Sensitivity analysis

In the RCT units (Supplementary Material: Supplementary Figure S7a), leave-one-out analysis demonstrated the robustness of the pooled effect size, which remained consistent despite fluctuations in heterogeneity. In the SAT units (Supplementary Material: Supplementary Figure S7b), the exclusion of a single outlying study led to the complete elimination of heterogeneity, identifying that study as the primary source of variance within this unit.

#### 3.4.3 Multiple comparisons of adverse events

A descriptive synthesis indicated that ITB therapy was generally well-tolerated in the BI population (Table 4). The most frequent PAEs included urinary retention, generalized muscle weakness, systemic hypotonia, constipation, and falls. But all PAEs were transient and successfully managed via pharmacological titration. CRAEs were primarily limited to catheter dislodgement and blockage. Notably, no permanent neurological injuries or treatment-related deaths occurred, supporting the preliminary safety profile of ITB for this population. Compared with the SCI population, the BI cohort showed a similar efficacy profile but a different pattern of CNS-related adverse events.

**TABLE 4:** Summary of adverse events of intrathecal baclofen in brain injury.

Study ID	Sample (N)	Pharmacological adverse events	Catheter-related adverse events
Meythale et al. (2001)	17	Headeche (1); urinary retention (5)	Catheter dislodgement (2)
Creamer et al. (2018)	24	Muscle weakness (4); seizure (1); peripheral edema (1); hypotension (1); urinary retention (3) Hypotonia (3); constipation (3); pain (2); fall (3)	Catheter dislodgement (1); catheter blockage (1)
Meythaler et al. (1999)	17	Drowsiness (1)	None

### 3.5 Cerebral palsy

Four studies (two RCTs, one SAT, and one mixed-design) investigated ITB treatment for CP. Due to the limited number of high-quality studies and substantial heterogeneity in design, sample size, and follow-up, a descriptive synthesis was performed instead of a quantitative meta-analysis.

#### 3.5.1 Spasticity

Across all included RCTs, ITB treatment significantly reduced spasticity scores, with qualitative descriptions further supporting clinical alleviation (Table 5). Similarly, SAT units confirmed significant post-treatment decreases in spasticity. Notably, site-specific improvements were documented in both upper and lower limbs, reinforcing the therapeutic efficacy of ITB across different body regions in the CP population.

**TABLE 5:** Summary of clinical outcomes for pediatric cerebral palsy population.

Study type	Study	Sample size (N)	Follow - up	Spasticity	Gross motor function	Caregiving	Key findings
Randomized controlled trials	Hoving et al. (2007)	27	6 h	Not reported	Not reported	Ease of care: 5.1 ± 0.8	Significantly reduced muscle spasticity (0.001 ≤ p ≤ 0.040)
							Improved ease of care (p = 0.001)
	Hoving et al. (2009)	12	6 Months	Not reported	Gross motor function measure: 1.3 ± 1.7	Ease of care: 3.9 ± 0.4	Effectively alleviates muscle spasticity
							Significantly improves gross motor function (p = 0.028)
							Reduces caregiving difficulty (p = 0.008)
	Gilmartin et al. (2000)	44	4 h	Ashworth scale: ↓1.22 ± 0.24	Not reported	Not reported	Effectively alleviates muscle spasticity (p < 0.001)
Single arm trials	Van Schaeybroeck et al. (2000)	6	12 Months	Ashworth scale: ↓0.5 (not report)	Not reported	Not reported	Significant reduction in muscle spasticity (P = 0.001)
							Functional improvement to some extent
							Improved quality of life
	Gilmartin et al. (2000)	41	6 Months	Ashworth scale: ↓1.33 ± 0.57 (lower limb)	Not reported	Not reported	Effectively alleviates muscle spasticity (p < 0.001)
				Ashworth scale: ↓0.74 ± 0.26 (upper limb)			

#### 3.5.2 Gross motor function

Quantitative analysis indicated improvements in gross motor function following ITB treatment, supported by qualitative reports of functional benefits in selected patients (Table 5). However, data regarding motor outcomes remain limited as most included studies did not incorporate these measures.

#### 3.5.3 Ease of care and quality of life

RCT units consistently demonstrated that ITB significantly reduced caregiving difficulty, with improvements reported across relevant scoring systems (Table 5). Regarding quality of life, available data indicated significant post-treatment enhancements in pediatric patients. However, evidence for these outcomes remains sparse, as they were not routinely reported across all included studies.

#### 3.5.4 Adverse events

Due to inconsistent reporting standards, a descriptive summary of AEs was performed (Table 6). In the CP population, PAEs were predominantly neuromuscular in nature, with systemic hypotonia being the most frequently reported symptom. CNS-related reactions included somnolence, headache, dizziness, and seizures. Gastrointestinal adverse events mainly included nausea and vomiting. CRAEs mainly involved catheter dislodgement and breakage, whereas pump pouch fluid accumulation and infections were the leading SRAEs. Although fatalities were documented in one study, they were adjudicated as independent of the pharmacological intervention.

**TABLE 6:** Summary of adverse events and complications across all populations.

Category	Common adverse events	Total cases	Incidence rate (%)	Relevant studies
Pharmacological adverse events	Drowsiness	26	20.77%	Hoving et al. (2007); Van Schaeybroeck et al. (2000); Gilmartin et al. (2000)
	Hypotonia	32	24.62%	Hoving et al. (2007); Gilmartin et al. (2000)
	Perspiration of hands and feet	1	0.77%	Hoving et al. (2007)
	Headache	14	10.77%	Gilmartin et al. (2000)
	Seizure	19	14.62%	
	Dizziness	6	4.62%	
	Sialorrhea	6	4.62%	
	Vomiting	14	10.77%	
	Nausea	17	13.8%	
	Symptoms of lowered cerebrospinal fluid pressure	12	9.23%	Hoving et al. (2007)
Catheter-related adverse events	Catheter breakage	3	1.54%	Hoving et al. (2007)
		20*	--	Gilmartin et al. (2000)
	Catheter dislodgement	20*	--	
Surgery-related adverse events	Pump pocket seroma	39*	--	
	Pump pocket infection	39*	--	
	Puncture-induced radiculopathy	12	9.23%	Hoving et al. (2007)
	Meningitis	2	1.54%	Gilmartin et al. (2000)
	Gastroenteritis	2	0.77%	Hoving et al. (2007)
Adverse events unrelated to the study	Death	2	0.77%	Gilmartin et al. (2000)

The data marked with * indicate the number of event occurrences, while all other unmarked data represent the number of patient cases.

## 4 Discussion

### 4.1 Therapeutic efficacy of ITB across different etiologies

The present findings support the clinical utility of ITB therapy in the management of severe spasticity across different neurological etiologies (Coffey et al., 1993; Albright et al., 1993; Hoving et al., 2009). This therapeutic effect is biologically plausible, given that baclofen acts predominantly at the spinal level through GABA_B_ receptor–mediated inhibition of excitatory neurotransmission and reduction of motor neuron excitability (Davidoff, 1985), thereby attenuating reflex hyperexcitability (Bowery, 1989; Trompetto et al., 2014). Moreover, intrathecal administration allows higher local drug exposure with lower systemic burden than oral therapy (Penn and Kroin, 1984), which may improve the therapeutic window and reduce treatment-limiting adverse effects (Krach, 2001). Together, these pharmacological properties provide a reasonable mechanistic explanation for the multidimensional benefits observed in the present analysis.

Despite the overall consistency in treatment efficacy, some variation in therapeutic response was observed across etiologies, suggesting that the clinical effects of ITB may be jointly influenced by the underlying pathophysiological state and functional background (Trompetto et al., 2014). In patients with SCI, the therapeutic trajectory after ITB appeared relatively stable, with sustained benefits observed across different dose levels. This finding suggests that, in some individuals with SCI, relatively low intrathecal doses may already be sufficient to achieve satisfactory antispastic effects, and that the traditionally assumed linear dose–response relationship may not always apply (Boster et al., 2016; Francisco et al., 2009). One possible explanation is the segmental spinal circuits responsible for baclofen responsiveness remain sufficiently intact below the lesion level (Adams and Hicks, 2005), although the underlying mechanisms remain to be clarified (Dietz and Sinkjaer, 2007). Notably, improvements in both spasticity severity and spasm frequency were maintained during both short-term and long-term follow-up, with no clear evidence of waning efficacy over time. To some extent, this finding supports the long-term stability of ITB in the management of SCI-related spasticity and may alleviate concerns regarding tolerance or diminishing treatment effects with prolonged use (Coffey et al., 1993; Boster et al., 2016). The absence of significant sex-based differences in the present analysis further suggests that treatment responsiveness may be relatively consistent within this population.

In patients with BI, the overall direction of antispastic outcomes was broadly consistent between RCTs and SATs, suggesting that the therapeutic effects of ITB may not be confined to highly controlled research settings but may also be reproducible in real-world clinical practice (Albright, 1996). Notably, subgroup analyses showed relatively consistent improvement in AS scores, whereas SFS outcomes appeared to vary by anatomical region (Rekand, 2010). In particular, responses in the upper limbs were more consistent, while those in the lower limbs showed greater variability. This pattern suggests that functional responses across different anatomical regions may not occur synchronously (Pandyan et al., 2005; Thibaut et al., 2013). A possible explanation is that lower-limb spasticity is more strongly influenced by prolonged positioning, weight-bearing status, and changes in peripheral sensory input (Trompetto et al., 2014; Lance, 1980). Another possibility is that intrathecal administration may result in differential drug exposure across spinal segments (Pandyan et al., 2005). However, these interpretations remain largely speculative and require further investigation (Boster et al., 2016). Taken together, these findings suggest that, in BI patients, reliance on a single spasticity scale may be insufficient to capture the full scope of clinical benefit (Pandyan et al., 2005; Rekand, 2010). A more comprehensive evaluation incorporating functional status, caregiving burden, and individualized treatment goals may therefore be necessary to guide patient-specific parameter adjustment (Boster et al., 2016; Francisco et al., 2009).

In children with CP, the available evidence was too limited and heterogeneous to support robust quantitative synthesis, and the findings were therefore interpreted primarily through descriptive qualitative analysis. Nevertheless, the overall direction of effect was broadly consistent across the available RCTs and single-arm trials, suggesting that ITB can still provide meaningful reduction in spasticity in this population. Importantly, however, alleviation of spasticity does not necessarily translate into parallel improvement in gross motor function, an issue that is particularly relevant in pediatric CP (Taricco et al., 2000). A possible explanation is that long-standing spasticity in these patients often coexists with muscle weakness, impaired selective motor control, established musculoskeletal deformities, and restricted motor development (Sanger et al., 2003; Gormley, 2001). As a result, even when neurogenic hypertonia is reduced, functional gains may remain constrained by multiple non-neural factors (Butler and Campbell, 2000; Gormley, 2001). In this context, the clinical value of ITB in children with CP may not be adequately captured by conventional motor outcomes alone, but may also be reflected in reduced caregiving burden, improved comfort, and better overall quality of care. For children with severe quadriplegic involvement, the primary treatment goal is often not restoration of independent ambulation, but rather improvement in positioning, transfers, hygiene-related care, and pressure injury prevention (Albright, 1996; Gormley, 2001). Accordingly, future studies that focus solely on spasticity scales or gross motor indices may still underestimate the real-world clinical value of ITB (Pandyan et al., 2005). High-quality studies are therefore needed to incorporate more patient-centered outcome measures, including functional performance, participation, and quality of life.

Overall, compared with oral antispastic agents, a major advantage of ITB lies in its delivery route, which provides high local bioavailability and allows continuous, dynamic, and individualized dose adjustment during long-term management. By following a “start low, go slow” strategy, clinicians may reduce harmful spasticity while preserving residual muscle tone that remains beneficial for posture maintenance, transfers, standing, or balance (Boster et al., 2016; Francisco et al., 2009). This point is clinically important, as excessive suppression of tone may paradoxically compromise residual motor capacity and postural stability (Rekand, 2010; Dietz and Sinkjaer, 2007). In the present study, most pharmacological adverse events occurred during the initial titration phase, suggesting that they were more likely related to enhanced central inhibitory effects and the process of dose adjustment rather than to an inherent and persistent safety limitation of ITB itself (Boster et al., 2016; Coffey et al., 2002). The definition of an optimal therapeutic window also appeared to differ across etiologies (Coffey et al., 2002). For example, patients with SCI may achieve satisfactory clinical responses at relatively low doses (Meythaler et al., 1992), whereas children with CP often require more cautious titration and may depend more heavily on postoperative rehabilitation and long-term follow-up to realize meaningful benefit (Gilmartin et al., 2000). Accordingly, the effective use of ITB does not rely solely on drug infusion itself, but on continuous multidisciplinary assessment of functional status, treatment goals, and adverse events, together with ongoing refinement of the treatment plan based on objective scales and feedback from both patients and caregivers. The clinical value of ITB is most fully realized only when efficacy, function, and safety are carefully balanced.

### 4.2 Safety profile and adverse events analysis

Although ITB therapy can provide multidimensional clinical benefits across a range of neurological disorders, its safety profile remains a major factor limiting broader clinical adoption and long-term management (Ridley and Rawlins, 2006). Previous studies have focused predominantly on PAEs, with comparatively less attention paid to non-pharmacological complications (Mossner et al., 2024). By separately evaluating PAEs and non-pharmacological adverse events, the present study highlights that, beyond drug-related toxicity, non-pharmacological complications also represent an important and non-negligible source of risk during ITB treatment and may substantially affect treatment continuity as well as overall clinical management (Brennan and Whittle, 2008).

#### 4.2.1 Pharmacological adverse events: Patterns and clinical implications

The pattern of PAEs also appeared to show some etiology-specific variation. In adults with SCI, constipation was the most frequently reported PAE, followed by headache, somnolence, and hypotension. Rather than indicating direct neurotoxicity, manifestations such as hypotension and constipation may more plausibly reflect baclofen-related modulation of spinal autonomic circuits and visceral function (Elbasiouny et al., 2010; Morozov and Nesterin, 2024), particularly through altered autonomic regulation and reduced visceral motility (Winter et al., 2018). By contrast, central nervous system symptoms such as somnolence and respiratory depression may suggest rostral spread of the drug after intrathecal administration, resulting in dose-dependent central inhibitory effects (Boster et al., 2016; Saulino et al., 2016).

In patients with BI, urinary retention, generalized muscle weakness, hypotonia, and falls were more prominent. One possible explanation is that, in the setting of pre-existing disruption of brainstem and cortical regulatory networks, these patients may be more susceptible to the rostral central effects of ITB (Suputtitada et al., 2024; Meythaler et al., 1999). In addition, whether the cerebrospinal fluid distribution pattern after intrathecal administration contributes to this phenomenon remains to be clarified. Therefore, in patients with BI, assessment should extend beyond routine monitoring of spasticity relief to include close (Pandyan et al., 2005), dynamic evaluation of postural control (Rekand, 2010), residual muscle strength, and fall risk, so as to avoid offsetting potential benefits through excessive suppression of muscle spasticity (Francisco and McGuire, 2012).

In children with CP, systemic hypotonia appeared to be particularly prominent and was often accompanied by central nervous system-related manifestations, such as somnolence, headache, dizziness, and seizures. This pattern may suggest that pediatric patients with CP are particularly susceptible to overtreatment during antispastic therapy with ITB (Masrour et al., 2024), potentially owing to the developmental immaturity of motor control networks and limited baseline functional reserve (Novak et al., 2020; Sanger et al., 2003). Nausea and vomiting were the predominant gastrointestinal PAEs observed in this population and may likewise be associated with baclofen-related effects on central regulatory pathways (Krach, 2001; Vitztum and Olney, 2000). These interpretations, however, remain largely speculative and warrant further mechanistic investigation. Nonetheless, the findings have important clinical implications, highlighting the need for especially cautious initiation, gradual dose titration, and close longitudinal monitoring when ITB is used in pediatric CP (Vitztum and Olney, 2000; Boster et al., 2016).

Nevertheless, although most of the PAEs described above appear to be at least partly dose-dependent and can often be effectively controlled through gradual titration and close monitoring (Bhimani, 2008; Suputtitada et al., 2024), serious events such as new-onset seizures or severe respiratory depression require immediate reassessment of ITB dosing and (Coffey et al., 2002), when necessary, dose reduction, temporary suspension, or discontinuation (Motta and Antonello, 2014). Individualized titration should therefore be considered a central element of ITB clinical management. Across patients with different etiologies, age groups, and functional status, optimizing ITB ultimately depends on achieving an appropriate balance between spasticity reduction and preservation of residual function (Butler and Campbell, 2000; Thibaut et al., 2013; Holtz et al., 2017).

#### 4.2.2 Non-pharmacological adverse events: patterns and clinical implications

Beyond PAEs, device- and procedure-related AEs constitute another important threat to the long-term safety of ITB therapy. In contrast to PAEs, these complications are often characterized by a more abrupt onset and greater potential for acute clinical deterioration (Romito et al., 2021; Kaye et al., 2025). In addition to interrupting drug delivery, they may precipitate acute baclofen withdrawal syndrome, a potentially life-threatening condition that can lead to rebound exacerbation of spasticity, rhabdomyolysis, hyperthermia, multiorgan dysfunction, and death (Kaye et al., 2025; Iqbal et al., 2026).

The long-term safety of ITB therefore depends not only on careful dose optimization, but also on a comprehensive risk-management strategy extending across the perioperative phase, longitudinal follow-up, and home care. Key components include standardized catheter implantation techniques, routine surveillance of catheter and pump integrity, strict aseptic refill protocols, and systematic education of patients and caregivers to enable early detection of pump alarms, infusion abnormalities, and prodromal features of withdrawal (Kaye et al., 2025; Cozzi et al., 2024).

In adults with SCI, pump-related adverse events, including pump stoppage, catheter kinking, fracture, pump pouch fluid accumulation and erosion, as well as pocket fluid collection and pump erosion, appeared to be relatively more frequent. The predominance of CRAEs and PRAEs in this population may be related to chronic mechanical stress associated with prolonged sitting, sensory loss, local skin vulnerability, and repetitive friction (Sezer et al., 2015; Kopp et al., 2024; Zakrasek et al., 2015). These factors may increase the risk of skin breakdown and other pocket-related complications (Zakrasek et al., 2015) and may also contribute to catheter instability or interruption of reliable drug delivery (Lake and Shah, 2019). Accordingly, long-term surveillance in SCI should extend beyond spasticity outcomes to include ongoing assessment of pump pocket skin integrity, pressure-related risk, and device stability.

In children with CP, catheter dislodgement, breakage, and perioperative infection appear to be of particular concern. Perioperative infections may reflect the complexity of surgical and postoperative care, together with limited local soft-tissue coverage (Ryan et al., 2024), whereas mechanical complications may be driven by ongoing changes in the relative configuration of the pump and catheter as the child grows (Held et al., 2026; Hagemann et al., 2020). These factors may, in turn, increase the risks of both infection and device failure (Albright and Ferson, 2006). In this context, body-device compatibility after implantation should not be regarded as fixed in pediatric patients, and long-term management should specifically account for the potential impact of growth on pump position, catheter tension, and overall system stability (Mosher et al., 2024). In addition, younger children and those with limited communication capacity may be less able to express postoperative discomfort or early warning symptoms, making early detection of complications more challenging (Mosher et al., 2024). Therefore, in pediatric CP, ITB management should focus not only on spasticity reduction, but also on perioperative wound management, infection surveillance, and structured long-term device monitoring.

In patients with BI, catheter dislodgement and blockage appeared to be the most prominent device-related complications. Although the overall incidence of hardware-related adverse events may not be disproportionately high, postoperative management in this population is often complicated by cognitive impairment (Silbert, 2024), behavioral dysregulation (Corrigan et al., 2023), and deficits in motor coordination (Capizzi et al., 2020), all of which may delay the recognition and management of complications. Reduced adherence and caregiving difficulties, for instance, may indirectly increase the likelihood of delayed identification of device malfunction or inadequate control of infection. Moreover, the frequent coexistence of impaired consciousness and seizures may further complicate postoperative surveillance and safety management (Frontera et al., 2024). These considerations underscore that ITB therapy in BI requires attention not only to the integrity and function of the device itself, but also to sustained multidisciplinary follow-up and close caregiver engagement.

Notably, although isolated deaths were reported in a small number of studies, the available evidence does not establish a direct causal link between these events and ITB therapy itself. In contrast, severe SRAEs such as bacterial meningitis deserve particular attention because they may not only necessitate treatment interruption, but also result in neurological deterioration and potentially fatal outcomes (Suputtitada et al., 2024; Saulino et al., 2016). This observation underscores that the safety of ITB is determined not only by the drug itself, but also by the quality of surgical technique, strict aseptic practice, and postoperative monitoring.

Overall, device- and procedure-related AEs constitute a major obstacle to the wider adoption of ITB and to the sustainability of long-term treatment (Boster et al., 2016). Their prevention and management should not follow a uniform model, but instead require stratified, individualized strategies based on disease etiology, age, and functional status (Ryan et al., 2024). In SCI, management should prioritize skin protection, pressure relief, and monitoring of device stability (Kumru et al., 2018). In pediatric CP, greater emphasis should be placed on perioperative infection prevention, longitudinal follow-up during growth, and ongoing assessment of device fit (Butler and Campbell, 2000). In BI, multidisciplinary care, caregiver engagement, and early identification of abnormal warning signs are particularly critical (Suputtitada et al., 2024). Integrating refined perioperative care, long-term device surveillance, and structured patient and caregiver education will be essential to minimizing these risks and supporting the safe long-term use of ITB.

### 4.3 Limitations and clinical implications

Despite providing robust evidence-based support, several limitations in this study warrant consideration for clinical practice. First, a notable disconnection exists between randomized evidence and real-world pharmacological optimization. While RCTs ensure internal validity, they often fail to simulate the long-term, dynamic dose titration and individualized maintenance phases that defines successful ITB therapy. This lack of longitudinal titration data may result in reported incidences of pharmacological AEs being higher than those observed in clinical settings where dose-response curves are meticulously adjusted.

Second, the quality and quantity of included evidence, particularly in certain subgroup analyses, were limited by small-study effects and earlier publication dates. From a safety pharmacology perspective, the limited sample sizes in certain subgroups may reduce the statistical power required to evaluate low-frequency but serious pharmacological toxicities. Furthermore, the relatively short follow-up periods (predominantly 3–12 months) are insufficient to comprehensively capture cumulative hardware-related risks and the potential long-term changes in GABA_B_ receptor sensitivity (e.g., pharmacological tolerance), which tend to manifest over years of continuous implantation (Schiess et al., 2020).

Finally, the inconsistency and fragmentation of outcome measures—especially the lack of standardized reporting on activities of daily livings and long-term quality of life in adult SCI and BI cohorts—restricts our ability to accurately quantify the non-linear pharmacodynamic relationship between molecular spasticity reduction and functional gain. Future research should prioritize longitudinal pharmacokinetic-pharmacodynamic (PK-PD) modeling and standardized functional assessments to better delineate the therapeutic window of ITB across diverse neurological populations.

## 5 Conclusion

This meta-analysis provides comprehensive evidence that ITB therapy delivers potent anti-spastic efficacy and functional improvements across diverse neurological etiologies, including SCI, BI, and CP. Our findings underscore that the pharmacotherapeutic success of ITB relies on a delicate balance between dose-dependent GABA_B_ receptor activation and the prevention of hardware-related complications. Notably, the distinct safety profiles observed across cohorts suggest that the pathophysiological microenvironment of the underlying condition significantly modulates the drug’s therapeutic window. These insights advocate for a transition from standardized protocols toward precision pharmacotherapy, characterized by etiology-specific dose titration and longitudinal monitoring of drug-receptor interactions. Future research integrating PK-PD modeling is essential to further optimize individualized treatment algorithms and enhance long-term functional outcomes in patients with refractory spasticity.

## Data Availability

The original contributions presented in the study are included in the article/Supplementary Material, further inquiries can be directed to the corresponding author.
