# Supplementary material for: Comparative pharmacotherapeutic efficacy and dose-related safety profiles of intrathecal baclofen: a systematic review and meta-analysis in neurological spasticity
Source: Front Pharmacol. 2026 Aug 24;17:1866389. doi: 10.3389/fphar.2026.1866389 (PMC13548089; doi:10.3389/fphar.2026.1866389)

Comparative Pharmacotherapeutic Efficacy and Dose-Related Safety Profiles of Intrathecal Baclofen: A Systematic Review and Meta Analysis in Neurological Spasticity

Haoyuan Chen [1,2], Honghui Lei [1,2], Yang Yu [1,2], Caizhi Ma [1,3,4], Fangyong Wang [1,2,3,4,5*]

[1] Department of Spine Surgery, Beijing Bo' ai Hospital, China Rehabilitation Research Center, Beijing, China.

[2] School of Rehabilitation, Capital Medical University, Beijing, China.

[3] School of Biological Science and Medical Engineering, Beihang University, Beijing, China.

[4] University of Health and Rehabilitation Sciences, Qingdao, Shandong, China.

[5] Rehabilitation Medicine Center, The Second Affiliated Hospital and Yuying Children' s Hospital, Wenzhou Medical University, Wenzhou, China.

*** Correspondence:** Fangyong Wang wfybeijing@163.com

Keywords: Intrathecal baclofen, Meta-analysis, Safety evaluation, Spasm frequency, Spasticity

Searching Strategies

1.1 Pubmed:

21 July 2025: (("baclofen"[Supplementary Concept] OR "baclofen"[All Fields] OR "baclofen"[MeSH Terms] OR "baclofen s"[All Fields] OR "baclofene"[All Fields]) AND "pump"[All Fields]) AND (randomized controlled trial [Filter])

4 August 2025: ("baclofen"[MeSH Terms] OR "baclofen"[All Fields] OR "baclofen s"[All Fields] OR "baclofene" [All Fields] OR "intrathecal baclofen"[All Fields]) AND ("pump"[All Fields] OR "intrathecal pump"[All Fields] OR"ITB pump"[All Fields]) AND (" randomized controlled trial"[Filter]OR "observational study"[Filter] OR "cohort study"[Filter]OR " case series"[Filter])

1.2 Web of Science:

21 July 2025: ("Baclofen" AND ("Infusion pump*") AND ("randomized" OR "RCT" OR "placebo")

4 August 2025: ("Baclofen" OR "Baclofen*") AND ("Infusion pump*" OR "drug delivery system " OR "intrathecal pump") AND ("randomized" OR "RCT" OR "placebo" OR "controlled trial" OR " observational study")

1.3 Cochrane Library:

21 July 2025: "intrathecal baclofen" OR "ITB therapy" OR "baclofen pump"

4 August 2025: ("intrathecal baclofen" OR "ITB therapy" OR "baclofen pump" OR "baclofen*" OR " spasticity treatment") AND ("pump" OR "intrathecal pump" OR "ITB pump" OR " continuous infusion" OR "drug delivery system")

1.4 Scopus:
4 August 2025: TITLE-ABS-KEY (("Baclofen" OR "Baclofen*") AND ("Infusion pump*" OR " drug delivery system" OR "intrathecal pump") AND ("randomized" OR "RCT" OR "placebo" OR " controlled trial" OR "observational study")

1.5 China Natinal Knowledge Infrastructure
21 July 2025: 主题=(("巴氯芬"OR"鞘内注射巴氯芬"OR "鞘内巴氯芬"OR"巴氯芬泵")AND("肌张力障碍"OR"痉挛状态")NOT 主题=("卡马西平"OR"原发性三叉神经痛")限定：学术期刊+学位论文
4 August 2025: 主题=(“巴氯芬”OR“巴氯芬泵”OR“鞘内注射巴氯芬”OR“鞘内巴氯芬”)AND(“痉挛”OR“肌肉痉挛”OR“痉挛程度”)来源类别：北大核心、CSCD 和 WJCI

Fig. S1. The quality of including literature

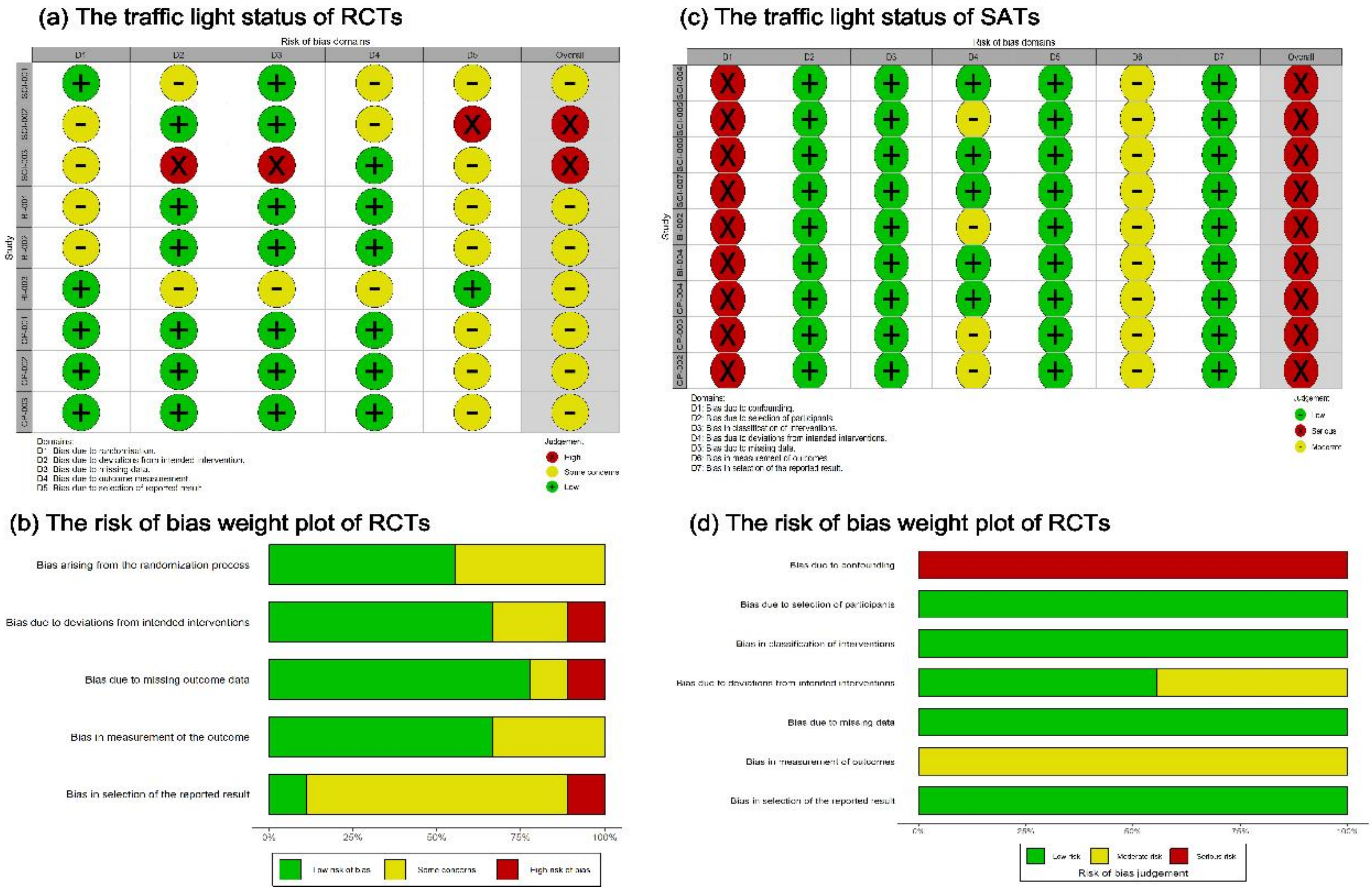

Fig. S2. The forest plot of Ashworth Scale reduction

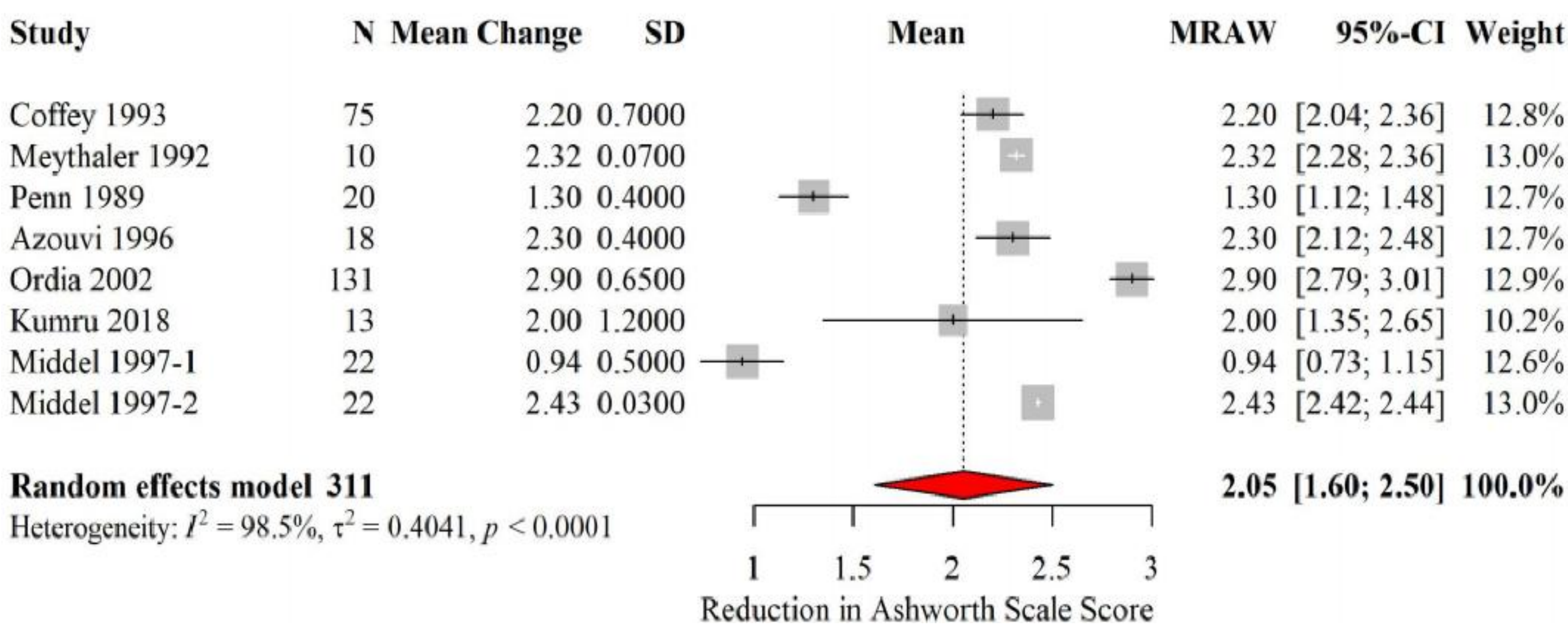

Fig. S3 Sensitivity analysis evaluating the influence of individual studies on the pooled effect size in Spinal Cord Injury

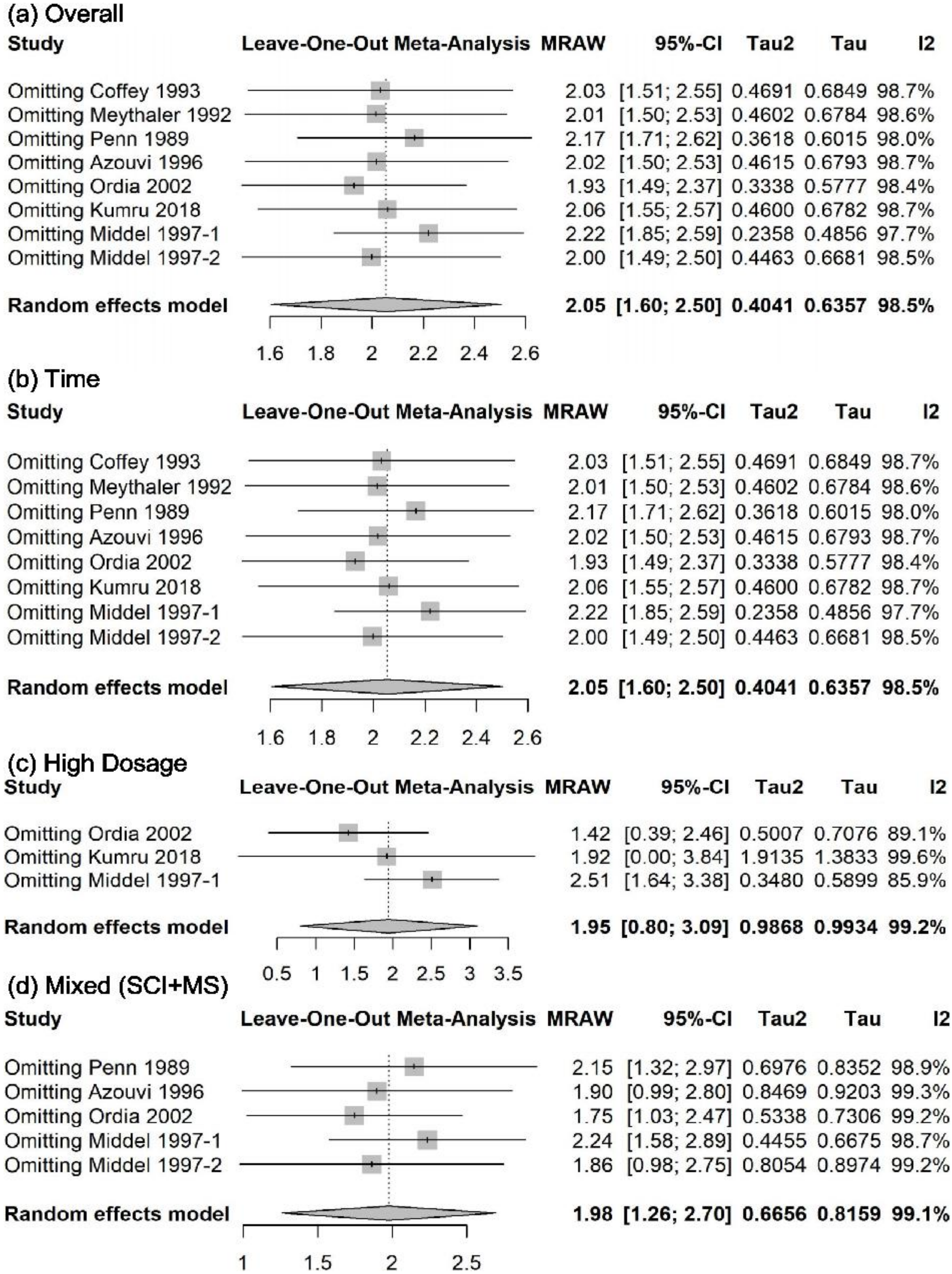

Table SI. Catheter-Related Adverse Events, Pump-Related Adverse Events and Surgery-Related Adverse Events

	No. of Studies	Total Patients	Pooled Incidence (%)	95% CI	I^2
Catheter-Related Adverse Events (CRAEs)					
Catheter Migration	3	171	3.51	1.58; 7.59	66.8%
Catheter Dislodgement	3	151	7.28	4.08; 12.67	92.7%
Catheter Kinking	5	191	11.52	7.71; 16.87	79.7%
Catheter Breakage	5	193	9.84	6.37; 14.92	78.0%
Pump-Related Adverse Events (PRAEs)					
Pump Flip	3	163	2.45	0.92; 6.35	22.4%
Programming Error	3	159	1.89	0.61; 5.68	46.4%
Pump Stoppage	3	52	5.77	1.87; 16.42	0.0%
Surgery-Related Adverse Events (SRAEs)					
Surgical Site Infection	3	159	3.14	1.31; 7.33	74.5%
Cerebrospinal Fluid Leak	4	173	3.47	1.57; 7.50	56.1%
Pump Pouch Erosion	3	163	3.68	1.66; 7.95	72.5%
Pump Pouch Fluid Accumulation	2	32	6.52	1.57; 21.81	0.0%

Fig. S4 The forest plot of Ashworth Scale reduction in randomized controlled trials and single arm trials in Brian Injury

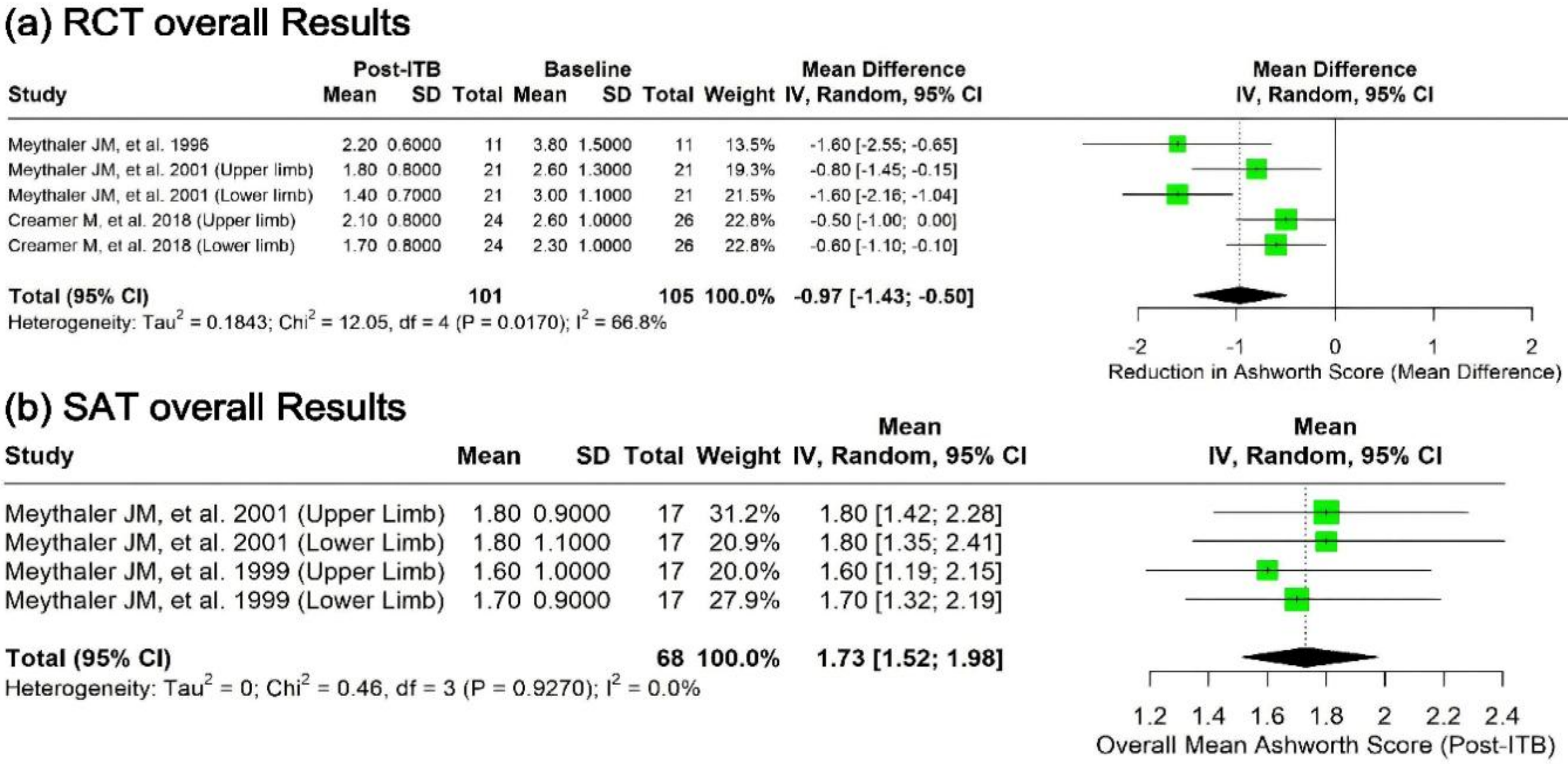

Fig. S5. Sensitivity analysis evaluating the influence of individual studies on the pooled effect size of Ashworth Scale

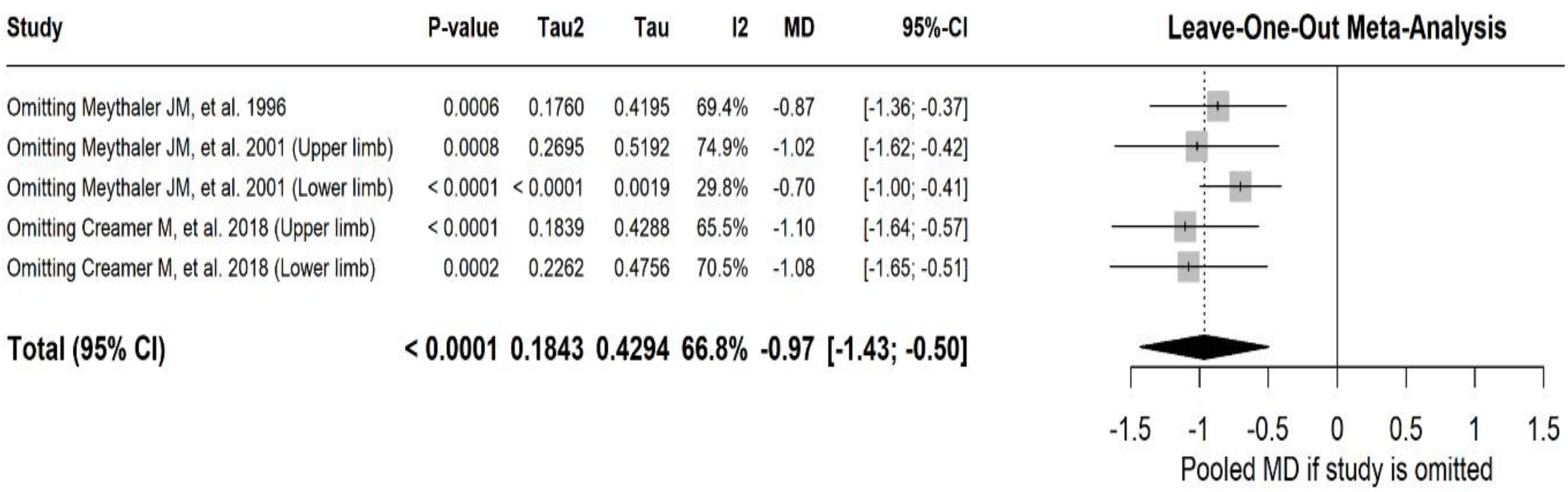

Fig. S6. The forest plot of Spasm Frequency Scale reduction in randomized controlled trials and single arm trials in Brian Injury

(a) RCT

Study	Post-ITB Mean	Post-ITB SD	Post-ITB Total	Baseline Mean	Baseline SD	Baseline Total	Weight	Mean Difference IV, Random, 95% CI
Meythaler JM, et al. 1996	1.00	1.0000	11	3.30	1.0000	11	30.3%	-2.30 [-3.14; -1.46]
Meythaler JM, et al. 2001 (Upper limb)	0.20	0.4600	21	0.73	1.1200	21	35.1%	-0.53 [-1.05; -0.01]
Meythaler JM, et al. 2001 (Lower limb)	0.10	0.3600	21	1.42	1.2600	21	34.5%	-1.32 [-1.88; -0.76]
**Total (95% CI)**			**53**			**53**	**100.0%**	**-1.34 [-2.32; -0.36]**

Heterogeneity: $Tau^2 = 0.6397$; $Chi^2 = 13.13$, df = 2 (P = 0.0014); $I^2 = 84.8\%$

(b) SAT

Study	Mean	SD	Total	Weight	Mean IV, Random, 95% CI
Meythaler 1999 (Upper)	0.71	0.8600	17	24.7%	0.71 [0.40; 1.26]
Meythaler 1999 (Lower)	1.36	0.9900	17	30.3%	1.36 [0.96; 1.92]
Meythaler 2001 (Upper)	0.39	0.9600	17	12.9%	0.39 [0.12; 1.26]
Meythaler 2001 (Lower)	0.53	0.2900	17	32.2%	0.53 [0.41; 0.69]
**Total (95% CI)**			**68**	**100.0%**	**0.73 [0.43; 1.24]**

Heterogeneity: $Tau^2 = 0.2082$; $Chi^2 = 19.35$, df = 3 (P = 0.0002); $I^2 = 84.5\%$

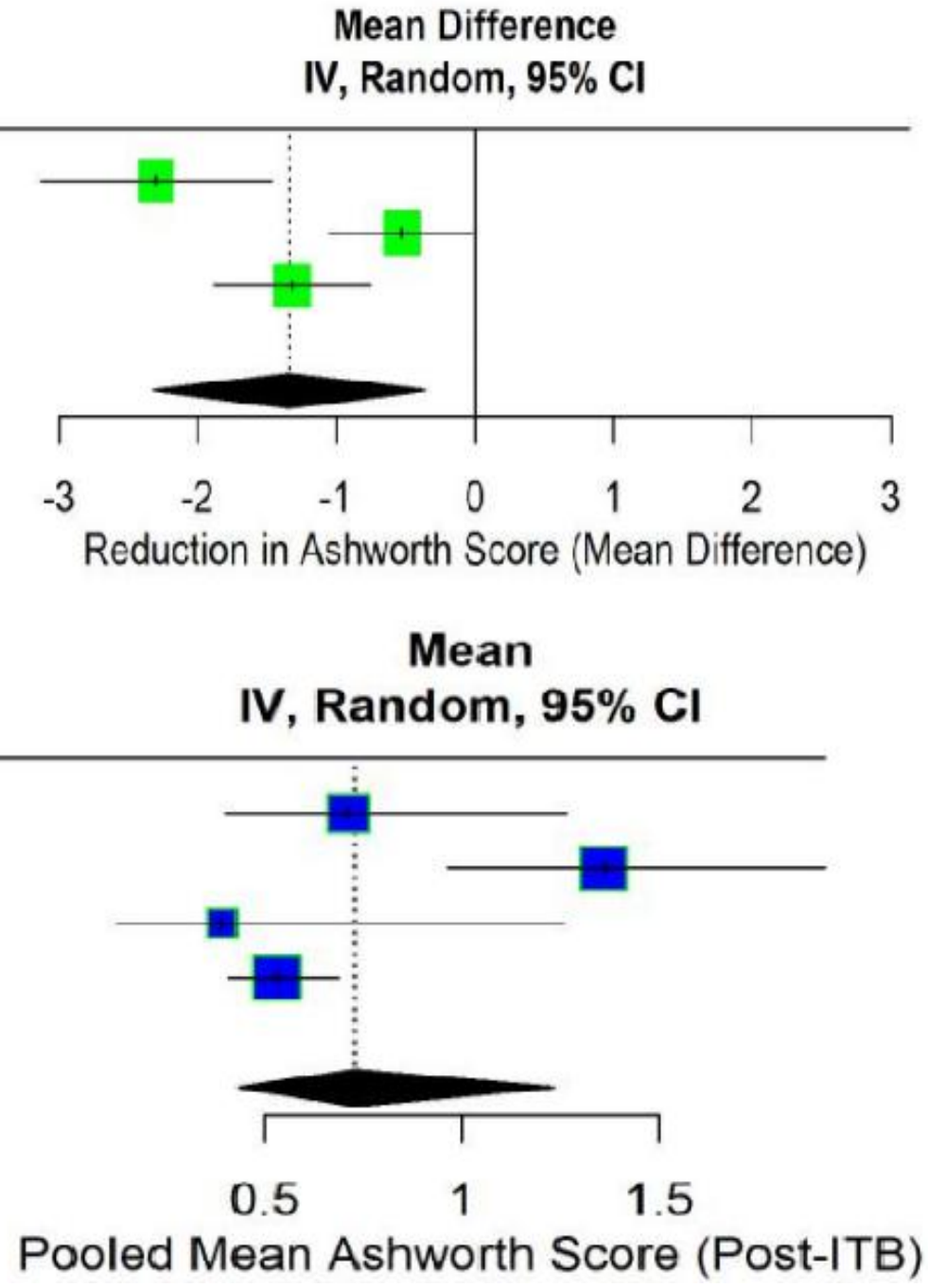

Fig. S7. Sensitivity analysis evaluating the influence of individual studies on the pooled effect size of Spasm Frequency Scale

(a) RCT

Study	P-value	Tau2	Tau	I2	MD	95%-CI
Omitting Meythaler 1996 （Overall）	0.0202	0.2363	0.4861	75.7%	-0.92	[-1.69; -0.14]
Omitting Meythaler 2001 （Lower Limb）	0.1178	1.4406	1.2003	92.0%	-1.38	[-3.12; 0.35]
Omitting Meythaler 2001 （upper Limb）	0.0003	0.3484	0.5903	72.6%	-1.76	[-2.71; -0.80]
**Total (95% CI)**	**0.0073**	**0.6397**	**0.7998**	**84.8%**	**-1.34**	**[-2.32; -0.36]**

(b) SAT

Study	Tau2	Tau	I2	Mean	95%-CI
Omitting Meythaler 1999 (Upper)	0.3400	0.5831	89.7%	0.72	[0.34; 1.51]
Omitting Meythaler 1999 (Lower)	0	0	0%	0.55	[0.44; 0.69]
Omitting Meythaler 2001 (Upper)	0.2207	0.4698	89.0%	0.80	[0.45; 1.43]
Omitting Meythaler 2001 (Lower)	0.2217	0.4709	69.9%	0.84	[0.44; 1.62]
**Total (95% CI)**	**0.2082**	**0.4563**	**84.5%**	**0.73**	**[0.43; 1.24]**

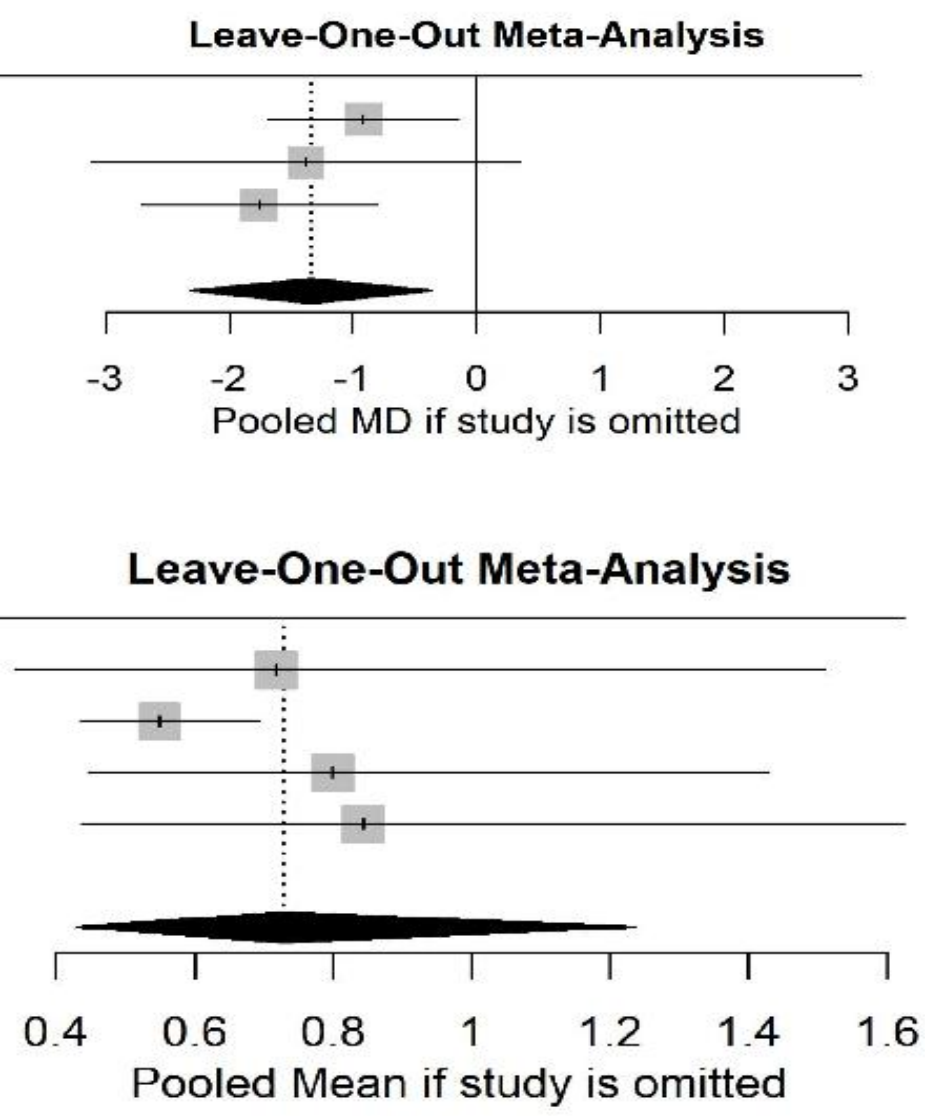

Supplement: Supplementary file 1 [file DataSheet1.pdf]
